# Checkpoint kinase 2 coordinates autophagy activation and Aurora kinase A degradation to regulate primary cilia for cell invasion

**DOI:** 10.1186/s12964-026-02953-6

**Published:** 2026-05-22

**Authors:** Chia-Yih Wang, Yu-Ying Chao, Ting-Yu Chen, Ruei-Ci Lin, Hui-Man Tsai, Hsiao-Han Huang, Zi-Rong Chen, Fu-I Lu, Yu-Chi Su, Bon-chu Chung

**Affiliations:** 1 Department of Cell Biology and Anatomy, College of Medicine, National Cheng Kung University, Tainan, 701 Taiwan; 2 Institute of Basic Medical Sciences, College of Medicine, National Cheng Kung University, Tainan, 701 Taiwan; 3 Institute of Molecular Biology, Academia Sinica, Taipei, 115 Taiwan; 4 National Center for Biomodels, National Institutes of Applied Research, Taipei, 115 Taiwan; 5 Department of Biotechnology and Bioindustry Sciences, College of Bioscience and Biotechnology, National Cheng Kung University, Tainan, 701 Taiwan; 6 The iEGG and Animal Biotechnology Center, National Chung Hsing University, Taichung, 402 Taiwan; 7 Graduate Institute of Biomedical Sciences, Neuroscience and Brain Disease Center, China Medical University, Taichung, 404 Taiwan

**Keywords:** Trophoblast invasion, Autophagy, Axoneme, Aurora A, Pancreatic cancer, Nutrient deprivation

## Abstract

**Background:**

Checkpoint kinase 2 (CHEK2) is a tumor suppressor that safeguards genome integrity in the nucleus. It is also localized at the mother centriole. But the role of CHEK2 in this structure remains unclear. The primary cilium acts as a sensory organelle that regulates various aspects of cell behavior. However, the connection between CHEK2 and primary cilia has not been elucidated.

**Methods:**

We investigated the role of CHEK2 in primary cilia regulation using both cultured cells and a zebrafish model. Pharmacological inhibition and genetic manipulation of CHEK2 were employed across systems, and rescue experiments were performed to validate the specificity of the findings. The primary cilia were observed using fluorescence or electron microscopy.

**Results:**

Here, we demonstrated that in response to nutrient deprivation, CHEK2 became activated to maintain primary cilia in both in vitro and in vivo loss-of-function and rescue studies. We found that CHEK2 maintained primary cilia by destabilizing Aurora Kinase A to prevent axoneme degradation and by activating AMPK to promote autophagy. Both pathways were essential for trophoblast ciliation, migration, and invasion. Moreover, in pancreatic ductal adenocarcinoma cells, glutamine deprivation activated CHEK2, which in turn coordinated autophagy induction and Aurora A degradation to sustain primary cilia and enhance invasive ability.

**Conclusions:**

In summary, our study uncovers a novel role of CHEK2 in mediating cell invasion under metabolic stress by promoting autophagy and stabilizing axoneme to maintain primary cilia.

**Graphical abstract:**

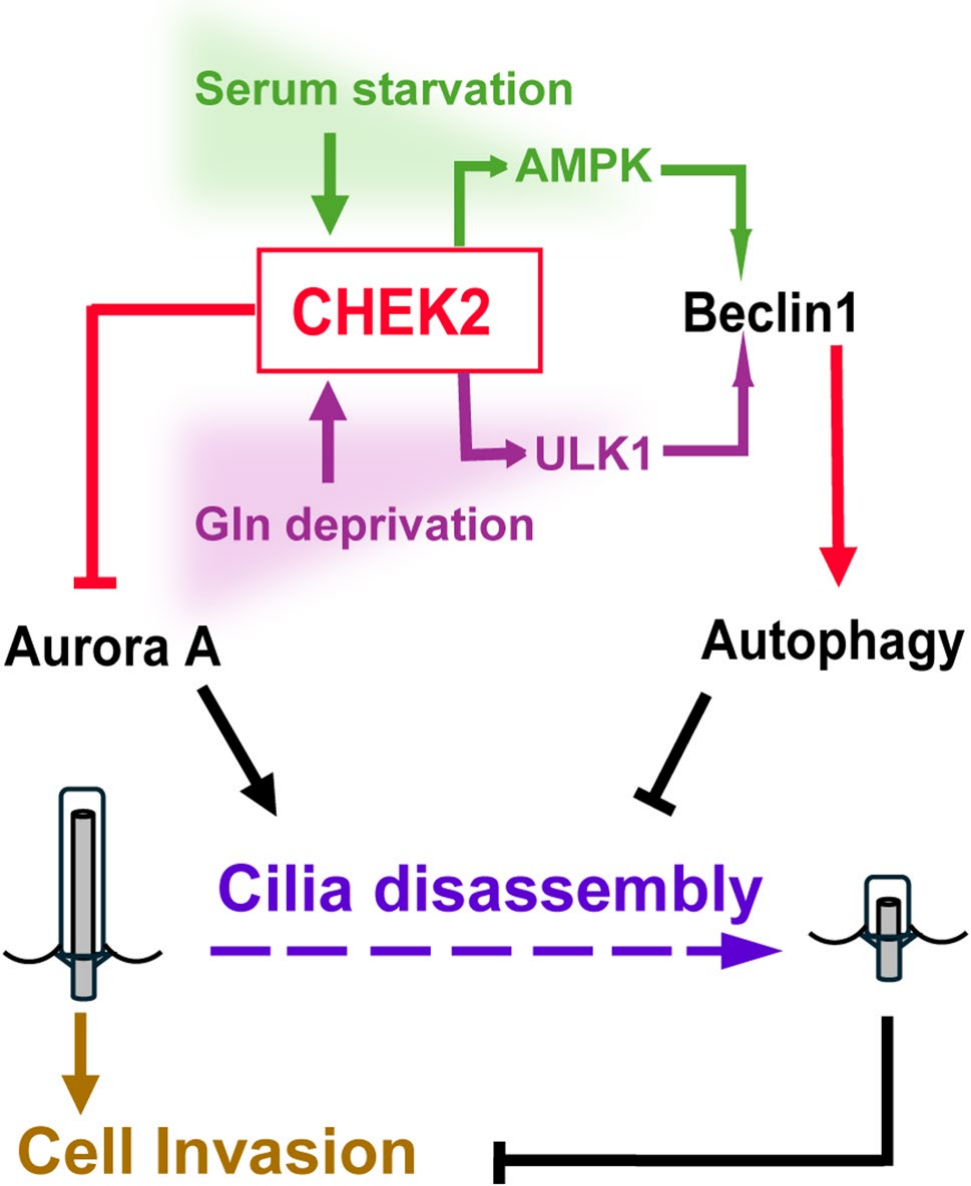

**Supplementary Information:**

The online version contains supplementary material available at 10.1186/s12964-026-02953-6.

## Background

Checkpoint kinase 2 (CHEK2) is a serine/threonine kinase with a role in DNA damage response and cell cycle regulation [1]. In response to cellular stress, CHEK2 is activated to preserve genomic integrity by orchestrating DNA repair mechanisms or inducing apoptosis [2]. CHEK2 functions also extend beyond these canonical roles, potentially influencing cellular structures and organelle dynamics [3]. Notably, CHEK2 has been implicated in centrosome regulation, where it induces centrosome amplification and disrupts their integrity upon prolonged replication stress in osteosarcoma [3–5].

Centrosomes serve as microtubule-organizing centers and are essential for the assembly of primary cilia [6]. Primary cilia are solitary, non-motile organelles that function as signaling hubs, mediating extracellular signal reception and transduction to regulate cell cycle progression, differentiation, and tissue morphogenesis [7]. Structurally, primary cilia consist of a microtubule-based axoneme and a specialized ciliary membrane that houses key signaling receptors [8].

Primary ciliogenesis is a tightly regulated process involving multiple steps. At the initiation step, Tau tubulin kinase-2 (TTBK2) is recruited to the mother centriole, where it phosphorylates CP110 and CEP83 to facilitate docking of pre-ciliary vesicles [9, 10]. Subsequent axoneme elongation is driven by microtubule extension and intraflagellar transport (IFT)-mediated bidirectional cargo trafficking [11]. Axonemal microtubules undergo several post-translational modifications, including acetylation and glutamylation, which are essential for ciliary stability and function [12, 13].

Primary cilia are closely linked to autophagy, particularly under conditions of metabolic stress such as serum starvation [14]. Autophagy is initiated through AMPK-dependent activation of ULK1, leading to LC3 cleavage and conjugation with phosphatidylethanolamine (LC3-II), which promotes autophagosome formation [15]. The fusion of autophagosomes with lysosomes facilitates cytoplasmic component degradation [16]. Notably, autophagy-mediated degradation of OFD1, a centrosomal protein that negatively regulates ciliogenesis, allows IFT20 retention and promotes primary cilia growth under nutrient deprivation [17–19].

Formation of primary cilia is tightly coordinated with the cell cycle. In quiescent cells (G0 phase), cilia are maintained, whereas re-entry into the cell cycle (G1/S transition) triggers ciliary disassembly [20]. TTBK2 stabilizes primary cilia, but during ciliary disassembly, it is ubiquitinated by E3 ubiquitin ligase HUWE1, leading to proteasomal degradation [21]. Additionally, axonemal microtubule deacetylation destabilizes the ciliary structure, promoting disassembly [22]. Aurora A kinase (Aurora A) plays a crucial role in this process by activating HDAC6, a tubulin deacetylase, to drive axoneme deacetylation and ciliary disassembly during cell cycle re-entry [23, 24]. Proper regulation of TTBK2 and Aurora A is therefore essential for cilia dynamics.

Primary cilia play a critical role in the function of trophoblasts, which are a specialized cell type in the placenta responsible for invading maternal uterine stroma to establish the maternal-fetal interface [25, 26]. Successful trophoblast invasion is essential for remodeling maternal spiral arteries, ensuring adequate blood flow and nutrient exchange between the mother and the fetus [27]. Defective ciliogenesis in trophoblasts has been associated with pregnancy-related complications such as preeclampsia and intrauterine growth restriction [28, 29].

Despite extensive studies on CHEK2 in DNA damage response, its role under metabolic stress remains poorly understood. Here, we demonstrated that CHEK2 is essential for maintaining primary cilia both in vitro and in vivo. Physiologically, CHEK2-mediated ciliary stability is crucial for trophoblast invasion and pancreatic cancer metastasis. Mechanistically, CHEK2 maintained primary cilia and preserved axonemal microtubule acetylation by promoting Aurora A degradation. Besides, CHEK2 also facilitated primary cilia through autophagy activation. Both events are critical for regulating primary cilia during metabolic stresses. Thus, our findings reveal a novel role for CHEK2 in sustaining primary cilia for cell invasion in response to distinct metabolic stresses.

## Materials and methods

### Cell culture

Human immortalized trophoblast HTR-8/SVneo (HTR8) cells and human pancreatic ductal adenocarcinoma (PANC-1) cells were maintained in Roswell Park Memorial Institute (RPMI)-1640 medium (Gibco, Grand Island, NY, USA). Human immortalized retinal pigment epithelial (RPE1), human alveolar basal epithelial (A549), and human kidney proximal tubular (HK-2) cells, as well as mouse Leydig progenitor (TM3) and mouse CNS catecholaminergic (CAD) cell lines, were cultured in Dulbecco’s Modified Eagle Medium (DMEM)/F-12 (Gibco). All culture media were supplemented with 10% fetal bovine serum (FBS; Gibco) and 1% sodium pyruvate (Gibco). Cells were maintained at 37 °C in a humidified incubator with 5% CO₂. Mycoplasma contamination was routinely monitored by immunofluorescence staining according to standard protocols.

### Zebrafish

Zebrafish (AB strain) was obtained from the Taiwan Zebrafish Resource Core Facility (TZCAS), originally from Zebrafish International Resource Center. Fish were cultured following the protocol of the zebrafish book [30]. The fertilized eggs were collected, and the chorion was removed by 1 mg/ml Protease (Sigma) digestion. The microinjection was performed at the one-cell stage using FemtoJet 4i (Eppendorf) equipped with a capillary and filament (Harvard, Part No. 30–0052). Phenotypes were examined using a dissecting microscope (Leica, Z16 APO). Zebrafish were handled under the guidance of the Institutional Animal Care and Use Committee (Protocol Number 106196) at National Cheng Kung University (Taiwan) and the 3R policy at the Ministry of Science and Technology (Taiwan).

### Morpholino (MO) design, RT-PCR, and mRNA synthesis

The morpholino (Gene tools) sequence used against *chek2* was the same as previously described [31]. Procedures for RNA extraction and RT-PCR from embryos were performed as previously described [32]. Full-length human chek2 (AF086904) was cloned into pCS2+, and chek2 mRNA was synthesized by SP6 polymerase provided by an in vitro transcription kit (Ambion) using Not1-digested plasmid as the template.

### Drug treatments

CHEK2 inhibitor II (CHEK2i, 220491) and MK-5108 (Aurora A inhibitor, S2770) were purchased from Selleckchem (Houston, USA). AMPK inhibitor (AMPKi, S7306), nocodazole (S2775), and cycloheximide (CHX, 66-81-9) were obtained from Sigma (St. Louis, MO, USA).

### Microtubule nucleation assay

Cells were treated with 25 µM nocodazole for 1 h to induce microtubule depolymerization. After treatment, cells were washed three times with PBS to remove residual nocodazole and then incubated in drug-free medium for the indicated periods. Cells were subsequently fixed with ice-cold methanol, followed by immunofluorescence staining to observe microtubule structures.

### Western blotting

Cells were lysed on ice for 10 min using CelLytic M cell lysis reagent (Sigma-Aldrich) supplemented with a protease inhibitor cocktail (Roche, Mannheim, Germany). Lysates were centrifuged at 13,300 rpm for 10 min at 4 °C, and the supernatants were collected. Protein concentrations were determined using the Bradford assay (Bio-Rad, Hercules, CA, USA). Lysates were mixed with 2X sample buffer and heated at 100 °C for 10 min. Proteins were separated by SDS-polyacrylamide gel electrophoresis (SDS-PAGE) and transferred onto polyvinylidene difluoride (PVDF) membranes at 20 V for 720 min at 4 °C. Membranes were washed with TBST, blocked with 3% bovine serum albumin in TBST for 1 h at room temperature, and incubated overnight at 4 °C with primary antibodies. After washing, membranes were incubated with horseradish peroxidase (HRP)-conjugated secondary antibodies for 1 h at room temperature. Protein signals were detected using enhanced chemiluminescence (ECL) reagents.

### Immunofluorescence microscopy

Cells were cultured on glass coverslips, fixed with ice-cold methanol at − 20 °C for 6 min, and blocked with 5% bovine serum albumin for 1 h at room temperature. Cells were incubated with primary antibodies overnight at 4 °C, followed by incubation with FITC- or Cy3-conjugated secondary antibodies (Invitrogen, Carlsbad, CA, USA) for 1 h at room temperature in the dark. Nuclei were counterstained with 0.1 µg/mL DAPI. After washing, coverslips were mounted using 50% glycerol in PBS. Images were acquired with an Axio Imager M2 microscope (Zeiss, Switzerland) and processed using ZEN Pro software (Zeiss). Primary cilia lengths were measured from z-stack projections using ZEN Pro analysis tools.

Zebrafish embryos were immunostained following an established procedure [33]. Briefly, the injected embryos were fixed in 4% paraformaldehyde with 0.25% TritonX-100 in 1X PBS overnight. The embryos then were incubated in blocking buffer (0.5% Triton-X-100 in 1X PBS containing 5% goat serum) for 4 h and then stained with anti-acetylated tubulin antibody (1:200, Sigma) in blocking buffer overnight for the second day. On the third day, the stained embryos were washed with wash buffer (0.5% TritonX-100 in 1X PBS) three times (30 min each). AlexaFlour 568-conjugated anti-mouse secondary antibody (1:200) was used to stain the embryo overnight. On the final day, the embryos were washed with wash buffer 3 times again and stained with 3 mM DAPI.

### Photography

Pictures were taken with a ZEISS LSM780 confocal microscope on zebrafish eye primordium region. The numbers of ciliated cells (cells that contain Acetylated-tubulin signal) in an area of 2500 µm^2^ were counted and the percentage of ciliated cells were calculated over the total number of cells labeled with DAPI using Image J (NIH).

### Antibodies

The following primary antibodies were used: anti-CHEK2 (#2662), anti-phospho-CHEK2 (Thr68; #2661), anti-E-cadherin (#3195), anti-vimentin (#5741), anti-Aurora A (#14475), anti-N-cadherin (#13116), anti-phospho-AMPK (Thr172; 40H9; #2535), anti-AMPK (#2532), anti-phospho-Beclin1 (Ser15; D4B7R; #84966), anti-Beclin1 (#3738), anti-LC3A/B (D3U4C, XP #12741), and anti-Ku70 (#4588) were purchased from Cell Signaling Technology (Beverly, MA, USA). Anti-IFT88 (13967-1-AP), anti-ARL13B (17711-1-AP), and anti-TTBK2 (15072-1-AP) antibodies were obtained from Proteintech (Chicago, IL, USA). Anti-CEP164 (NBP1-81445) was purchased from Novus (Littleton, CO, USA). Anti-β-actin (AC-15; GTX26276) was purchased from GeneTex (Irvine, CA, USA). Anti-acetylated tubulin (T6793) and anti-α-tubulin (A11126) were obtained from Sigma-Aldrich. Anti-CP110 (ab99338) was purchased from Abcam (Cambridge, UK).

### RNA interference (RNAi)

For depletion of CHEK2, IFT88, and CEP164 in HTR8, RPE1, and PANC-1 cells, the following siRNA sequences were used: - siCHEK2: 5’-aagaaccugaggaccaagaac [dT][dT]-3’, - siIFT88: 5’-cgacuaagugccagacucauu [dT][dT]-3’, - siCEP164: 5’-caggugacauuuacuauuuca [dT][dT]-3’. For BECN1 depletion, SMARTpool siGENOME siRNAs were used with the following sequences: - siBECN1: 5’-ccaaccagcuuaagaggaa-3’; 5’-gaagugaacauguggauca-3’; 5’-gagcagugccuuugaagaa-3’; 5’-ccaagaaggacucggguca-3’. The nucleotide sequences of the siRNA targeting regions of CHEK2 mRNA in human and mouse are identical.

A scrambled siRNA (5’-gaucauacgugcgaucaga [dT][dT]-3’) was used as a control (Sigma, St. Louis, MO, USA).

For transfections, 10 µL Lipofectamine 2000 (Invitrogen) was mixed with 500 µL Opti-MEM (Life Technologies) for 5 min, then combined with 2 µL of 100 µM siRNA diluted in 500 µL Opti-MEM. After a 20-minute incubation at room temperature, the mixture was added to cells in 1 mL DMEM/F12 medium (final siRNA concentration 100 nM). Cells were harvested 72 h post-transfection.

### Generation of CHEK2 knockout RPE1 cells

Stable CHEK2-knockout RPE1 cells were generated according to the established protocol (RNAi core, Academia Sinica, Taipei, Taiwan). Briefly, CRISPR/Cas9-mediated targeting was achieved by co-transfecting RPE1 cells with a surrogate reporter plasmid that expresses both GFP and mCherry when indel mutation occurs and an all-in-one plasmid that contains both Cas9 and a guide RNA targeting *CHEK2* (5’-accgucucgggagucggauguug-3’). The GFP^+^mCherry cells were sorted and diluted to single cells. Eleven clones grew up and their *CHEK2* gene was sequenced. Five clones containing indel mutation were further assessed for CHEK2 production by Western blot analysis.

### Transmission electron microscopy

For serial section TEM, cells were cultured on Aclar film (Electron Microscopy Sciences) placed in 12-well plates and fixed with 2.5% glutaraldehyde in 0.1 M cacodylate buffer (pH 7.2) for 20 min at room temperature. Following fixation, cells were rinsed in cacodylate buffer and post-fixed in 1% osmium tetroxide for 30 min. After triple rinsing with distilled water, samples were stained with 1% uranyl acetate for 30 min and rinsed again. Dehydration was performed through a graded ethanol series (50%, 70%, 95%, and 100%) for 5 min each. Samples were infiltrated with Epon resin and embedded in rubber molds, followed by polymerization at 65 °C for 48 h. Ultrathin serial Sect.  (70 nm) were cut using a diamond knife on an ultramicrotome (Leica EM UC7), collected on copper mesh grids, and stained with 4% uranyl acetate for 3 min and lead citrate for 10 min. Sections were imaged using a Tecnai G2 Spirit TWIN transmission electron microscope (Thermo Fisher Scientific) operated at 120 kV. Images were captured with a Gatan CCD camera (SC1000, 4008 × 2672 pixels) and processed using Gatan Digital Micrograph software.

### Flow cytometry

Cell cycle distribution was assessed using fluorescence-activated cell sorting (FACS) following a modified protocol [3]. Briefly, cells were harvested by trypsinization, washed, and re-suspended in phosphate-buffered saline (PBS). After centrifugation at 1,000 rpm for 5 min, cells were washed again and re-suspended in PBS containing 1 mM EDTA. The cell pellet was then fixed in 70% ice-cold ethanol overnight at 4 °C. Fixed cells were washed with PBS-E and stained with propidium iodide (Southern Biotech, Birmingham, AL) for 1 h at room temperature. DNA content was analyzed using a FACScan flow cytometer (Becton-Dickinson, San Diego, CA), and cell cycle profiles were processed using Kaluza software (Beckman Coulter, Brea, CA).

### Transwell invasion and migration assays

In vitro migration and invasion assays were performed using transwell inserts (8 μm pore size; Falcon #303597) in 24-well plates. Medium containing 10% FBS was placed in the lower chamber as a chemoattractant. For invasion assays, 100 µL of Matrigel (Corning) was applied to the upper surface of the insert and allowed to solidify. Cells (3 × 10⁴ in 100 µL serum-free medium) were seeded into the upper chamber and incubated at 37 °C with 5% CO₂ for 12 h. Then, the Matrigel was removed and inserts were fixed with 100% methanol for 5 min, air-dried, stained with 20% Giemsa (Merck #109204) for 2 h, rinsed with water, and carefully wiped to remove non-migrated or non-invaded cells. Cells on the lower surface were visualized and counted in randomly selected fields. Those cells that moved through the porous membrane were counted as migrating cells, and those that migrated through both the membrane and the Matrigel were invading cells.

### Whole-mount in situ hybridization and immunostaining

The procedures of whole mount in situ hybridization were done as described before [34], and the protocol is also available in the ZFIN (https://zfin.org/ZFIN/Methods/ThisseProtocol.html). The primer sequences used for creating the probe are 5’-TTAAGTGTGTGTGTCCAGAA-3’(Forward) and 5’-TCAAGGCCCGGGTTTCCTCTTGG-3’(Reverse).

### Statistical analysis

All experiments were performed with at least three independent biological replicates. Data are presented as mean ± standard deviation (SD) based on more than 100 cells per group. Comparisons between two groups were performed using unpaired two-tailed Student’s *t*-tests, while comparisons among multiple groups were analyzed using one-way ANOVA. A *p*-value < 0.05 was considered statistically significant.

## Results

### CHEK2 promotes ciliation under serum deprivation via its kinase activity

**Fig. 1:**
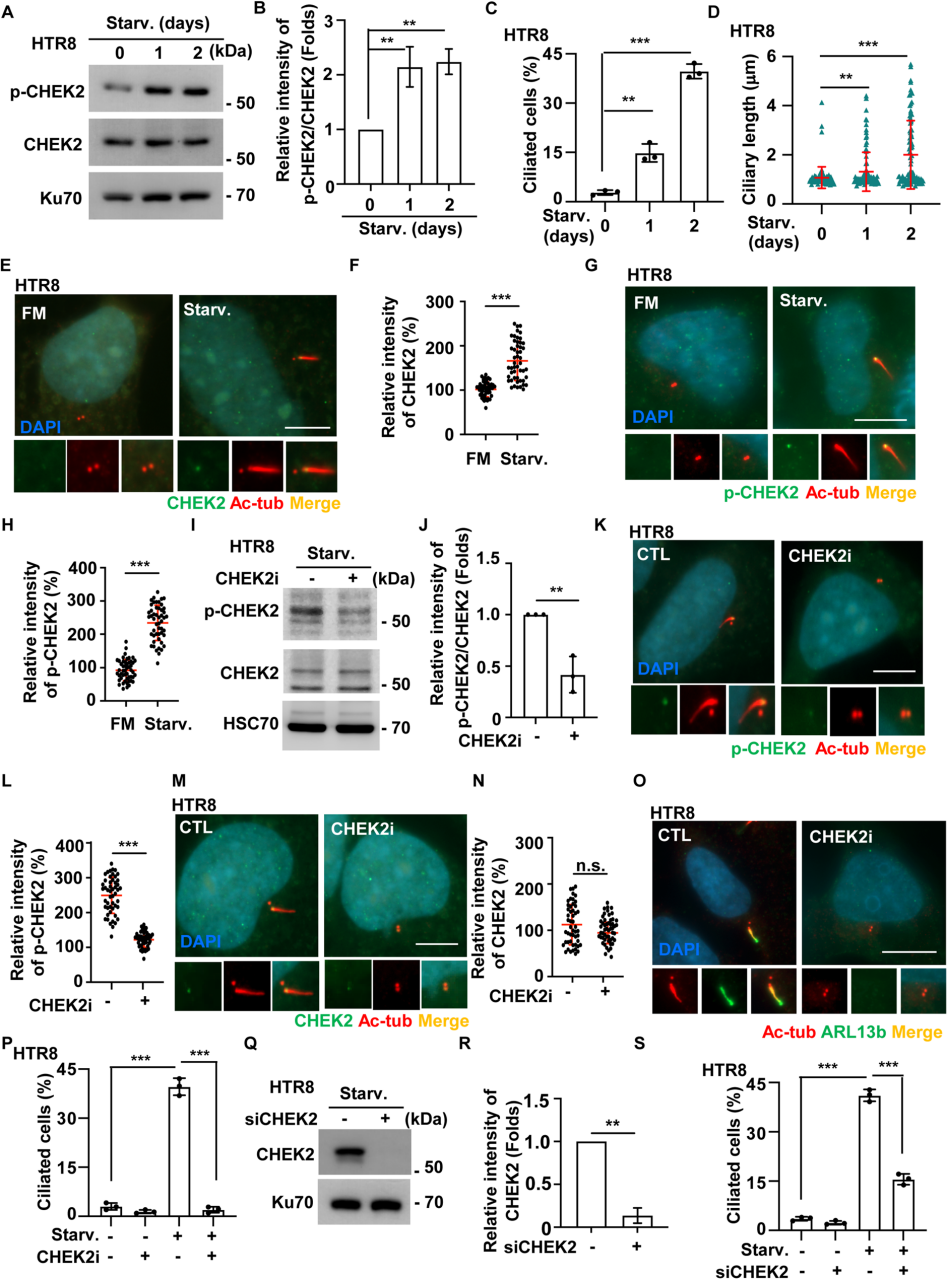
Serum starvation leads to CHEK2 phosphorylation that regulates primary cilia.** A**-**B** CHEK2 was phosphorylated in trophoblast HTR-8 cells upon serum starvation. (A) Extracts of HTR-8 cells under serum starvation (Starv.) for 0, 1, and 2 days were analyzed by Western blotting with antibodies against phosphorylated CHEK2 at Thr68 (p-CHEK2), CHEK2, and Ku70 (internal control). **B** Quantitative results for the relative intensity of p-CHEK2 to CHEK2 in (A). **C**-**D** Serum starvation induced primary ciliation. Quantitative results of (**C**) ciliated HTR-8 cells and (**D**) the length of primary cilia upon serum starvation for 0, 1, and 2 days. **E**-**H** (**E**-**F**) Total CHEK2 and (**G**-**H**) p-CHEK2 are localized to the base of primary cilia. CHEK2 of HTR-8 cells cultured in full-medium (FM) or serum-starved medium (Starv.) were observed by immunofluorescence staining with antibodies against CHEK2, p-CHEK2 and acetylated tubulin (Ac-tub). DNA were stained with DAPI. Scale bar: 10 μm. **F **and **H** Quantitative results for the relative intensity of CHEK2 and p-CHEK2 in (**E**) and (**G**), respectively. **I**-**N** CHEK2 inhibitor (CHEK2i) reduced CHEK2 activation upon serum starvation. **I**-**J** CHEK2i inhibited CHEK2 phosphorylation. **I** Extracts of HTR-8 cells in the presence or absence of CHEK2i under serum starvation were analyzed by Western blotting with antibodies against p-CHEK2, CHEK2, and HSC70. **J** Quantitative results for the relative intensity of p-CHEK2 to CHEK2 in **I**. **K**-**N** Reduction of phosphorylated CHEK2 at the ciliary base upon CHEK2i treatment. **K**-**L** Total CHEK2 and (**M**-**N**) phosphorylated p-CHEK2 of HTR-8 cells in the absence (CTL) or presence of CHEK2i under serum deprivation were observed by immunofluorescence staining with antibodies against CHEK2, p-CHEK2 and acetylated tubulin (Ac-tub). DNA were stained with DAPI. Scale bar: 10 μm. **O**-**S** Requirement of CHEK2 for primary ciliogenesis. **O**-**P** Inhibition of CHEK2 reduced primary cilia under serum starvation. **O** The primary cilia were reduced in CHEK2-inhibited HTR-8 cells under serum starvation. The axoneme (acetylated tubulin, Ac-tub) and ciliary membrane (ARL13b) were observed by immunofluorescence staining. DNA were stained with DAPI. Scale bars: 10 μm. **P** Quantitative results of ciliated HTR-8 cells. **Q**-**S** Depletion of CHEK2 reduced primary cilia under serum starvation. **Q** Western blot showing efficient depletion of CHEK2 by siCHEK2. **R** Quantitative results for the relative intensity of CHEK2 in **Q**. **S** Quantitative results of ciliated HTR-8 cells in the presence or absence of siCHEK2. **p < 0.01, ***p < 0.001, n.s.: no significance. These results are mean ± SD from three independent experiments. At least 300 cells were counted in each individual group

CHEK2 is located both at the nucleus and the centrosome [4, 35], and specifically at the mother centriole [3]; however, its role at the mother centriole remains poorly understood. Since the mother centriole functions as the basal body to grow primary cilia [36], we tested whether CHEK2 has a role in primary cilia using human trophoblast HTR-8/SVneo (HTR-8). HTR-8 cells were cultured in serum‑deprived medium for 24–48 h to induce the formation of primary cilia, and cell cycle profiles were analyzed by flow cytometry. Serum deprivation as well as hydroxyurea (HU) treatment (positive control) increased the proportion of cells in the G0/G1 phase, confirming cell cycle arrest under these conditions (Supplementary Fig. S1A-B). Upon serum deprivation, CHEK2 became phosphorylated (Fig. 1A-B), and both the proportion of ciliated cells and primary cilium length were increased with time (Fig. 1C-D). In starved primary cilia-bearing HTR-8 cells, total (Fig. 1E-F) and phosphorylated CHEK2 (Fig. 1G-H) signals were significantly enriched at the basal body compared with non‑ciliated cells cultured in full medium. Thus, CHEK2 is activated and recruited to the basal body during serum deprivation.

Then, we examined whether activated CHEK2 is required for primary ciliogenesis. When CHEK2 activity was inhibited by its selective inhibitor (CHEK2i), the levels of phosphorylated CHEK2 were reduced (Fig. 1I-J). Immunofluorescence staining showed that phosphorylated (Fig. 1K-L), but not total (Fig. 1M-N), CHEK2 signals at the basal body were markedly decreased in CHEK2i‑treated cells. In addition, the axoneme marked by acetylated tubulin and the ciliary membrane ARL13b were largely lost (Fig. 1O-P) when CHEK2 was inhibited. This was further supported by the loss of the intraflagellar transporter IFT88 signal (Supplementary Fig. S1C), while the distal appendages of the mother centriole marked by CEP164 were unaffected (Supplementary Fig. 1D-E). When HTR-8 cells were depleted of CHEK2 using siRNA (Fig. 1Q-R), the proportions of ciliated cells were reduced upon starvation (Fig. 1S), supporting that CHEK2 induces primary cilia.

Previous studies showed that CHEK2 activation induces centriole amplification [3], and CHEK2 is activated under serum starvation. We therefore examined whether centriole amplification is induced under these conditions. Starvation increased the proportion of cells with non-duplicated centrioles (1–2 centrioles), while reducing the proportion of cells with duplicated centrioles (3–4 centrioles) (Supplementary Fig. S1F). However, the proportion of cells exhibiting centriole amplification (more than four centrioles) was not affected. Furthermore, no significant difference was observed between control and CHEK2 inhibitor-treated cells (Supplementary Fig. S1F), indicating that CHEK2 activation does not promote centriole amplification, and that its inhibition does not alter centriole copy number under starvation. Collectively, these findings demonstrate that CHEK2 localizes to the basal body and is required for primary ciliogenesis under starvation conditions.

To determine whether this phenotype was not restricted to specific cells, we examined other cell lines. Indeed, inhibition of CHEK2 during serum deprivation reduced the proportion of ciliated human retinal pigmented epithelial (RPE-1), mouse fibroblast (NIH3T3) cells, mouse CNS catecholaminergic (CAD), mouse Leydig progenitor (TM3), human kidney proximal tubular (HK-2), human alveolar basal epithelial adenocarcinoma (A549), and human pancreatic ductal adenocarcinoma (PANC-1) cells (Supplementary Fig. S1G-M). Efficient depletion of CHEK2 by siRNA also led to a reduction of ciliated RPE-1 (Supplementary Fig. S1N-P) and NIH-3T3 cells (Supplementary Fig. S1Q-R). Thus, serum starvation activates CHEK2, which induces primary ciliation of many types of cells.

**Fig. 2:**
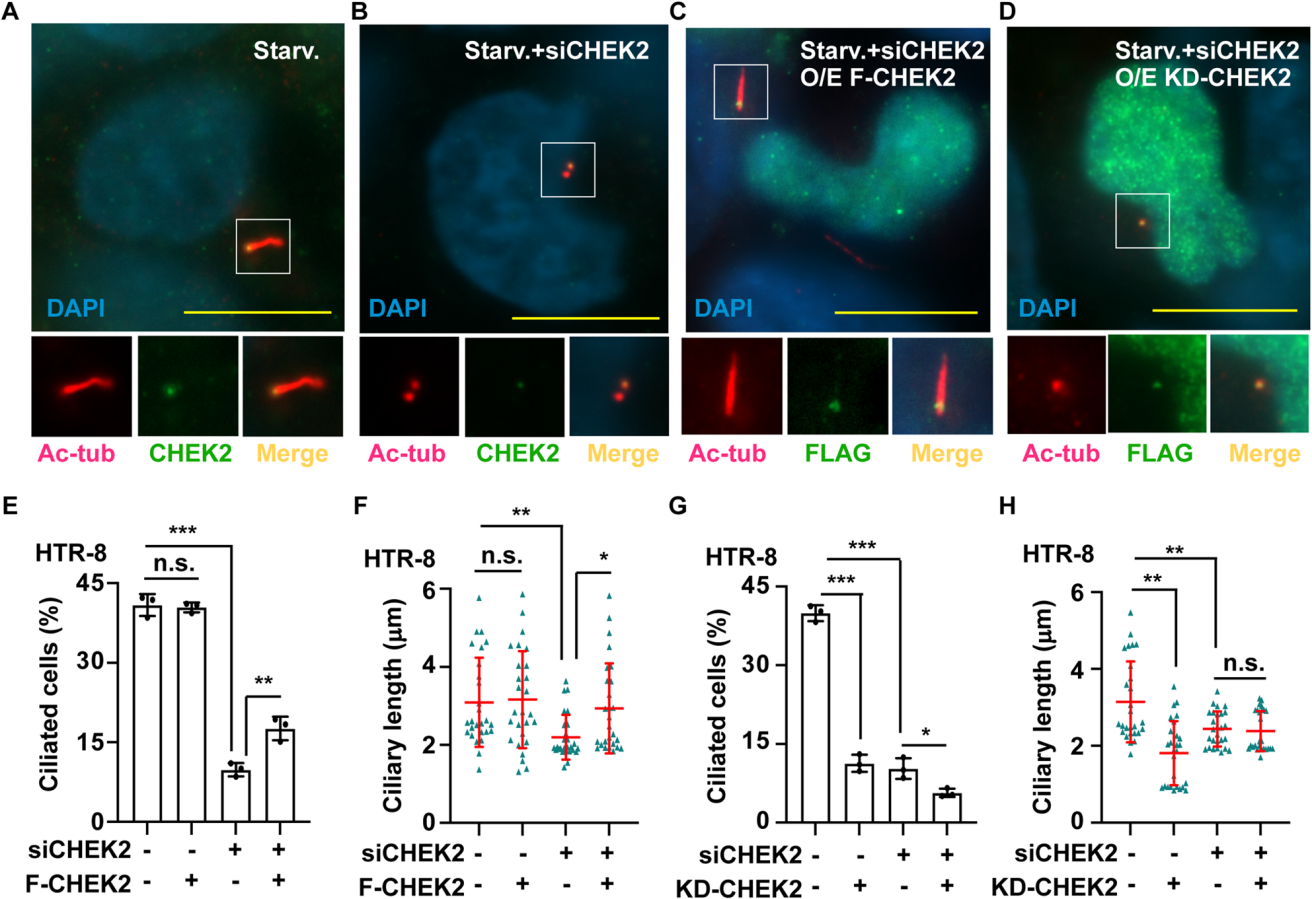
CHEK2 regulates primary cilia via its kinase activity.** A**-**D** CHEK2 is localized at the base of primary cilia. HTR-8 cells were serum-starved for 24 h and then examined for (**A**–**B**) endogenous CHEK2 under (**A**) control (Starv.), **B** CHEK2-deficient (siCHEK2), exogenous (**C**) FLAG-tagged wild-type CHEK2 (O/E F-CHEK2), or (**D**) kinase-dead CHEK2 (O/E KD-CHEK2) in CHEK2-deficient cells. Signals were detected by immunofluorescence staining with antibodies against acetylated tubulin (Ac-tub), CHEK2, and FLAG. DNA were stained with DAPI. Scale bar: 10 μm. **E**-**F** Overexpression of wild-type CHEK2 rescued the defect of (**E**) ciliated cells and (**F**) the length of primary cilia in scramble control or CHEK2-deficient (siCHEK2) HTR-8 cells with or without overexpressing FLAG-tagged CHEK2 (F-CHEK2). **G**-**H** Overexpression of kinase-dead CHEK2 inhibited primary cilia. Quantitative results of (**G**) ciliated cells and (H) the length of primary cilia in scramble control or CHEK2-deficient (siCHEK2) HTR-8 cells with or without overexpressing kinase-dead CHEK2 (KD-CHEK2). n.s.: no significance, *p < 0.05, **p < 0.01, ***p < 0.001. These results are mean ± SD from three independent experiments

To ensure the function of CHEK2 further, rescue experiments were conducted. Overexpression of FLAG-tagged CHEK2 (F-CHEK2) rescued the proportion of ciliated CHEK2-deficient HTR-8 (Fig. 2A-E) and RPE-1 cells (Supplementary Fig. S2A), and ciliary length was restored (Fig. 2F). To test whether the kinase activity of CHEK2 is required, the kinase-dead CHEK2 (KD-CHEK2) was overexpressed. It could not restore or even suppress the proportion of ciliated HTR-8 cells and the length of primary cilia (Fig. 2D and G-H) after CHEK2 depletion, implying a dominant-negative effect of KD-CHEK2 on primary cilia. Interestingly, both exogenous wild-type CHEK2 (F-CHEK2) and KD-CHEK2 localized to the basal body (Fig. 2C-D), suggesting that CHEK2 targeting to the cilia is independent of its kinase activity.

In addition to transient depletion using siRNA, we generated CHEK2 knockout (KO) RPE-1 cells using CRISPR-Cas9. These KO clones did not express any CHEK2 (Supplementary Fig. S2B), and the proportions of their ciliated cells were reduced when compared to the parental WT RPE-1 cells (Supplementary Fig. S2C). Overexpression of F-CHEK2, but not KD-CHEK2 rescued the primary cilia in 5C5 and 6C3 KO cells (Supplementary Fig. S2D-F). Interestingly, KD-CHEK2 further suppressed primary cilia of parental RPE-1 cells (Supplementary Fig. S2E). Taken together, our data show that the kinase activity of CHEK2 is required to regulate primary ciliogenesis upon serum deprivation.

### CHEK2 regulates primary cilia formation in zebrafish embryos

**Fig. 3:**
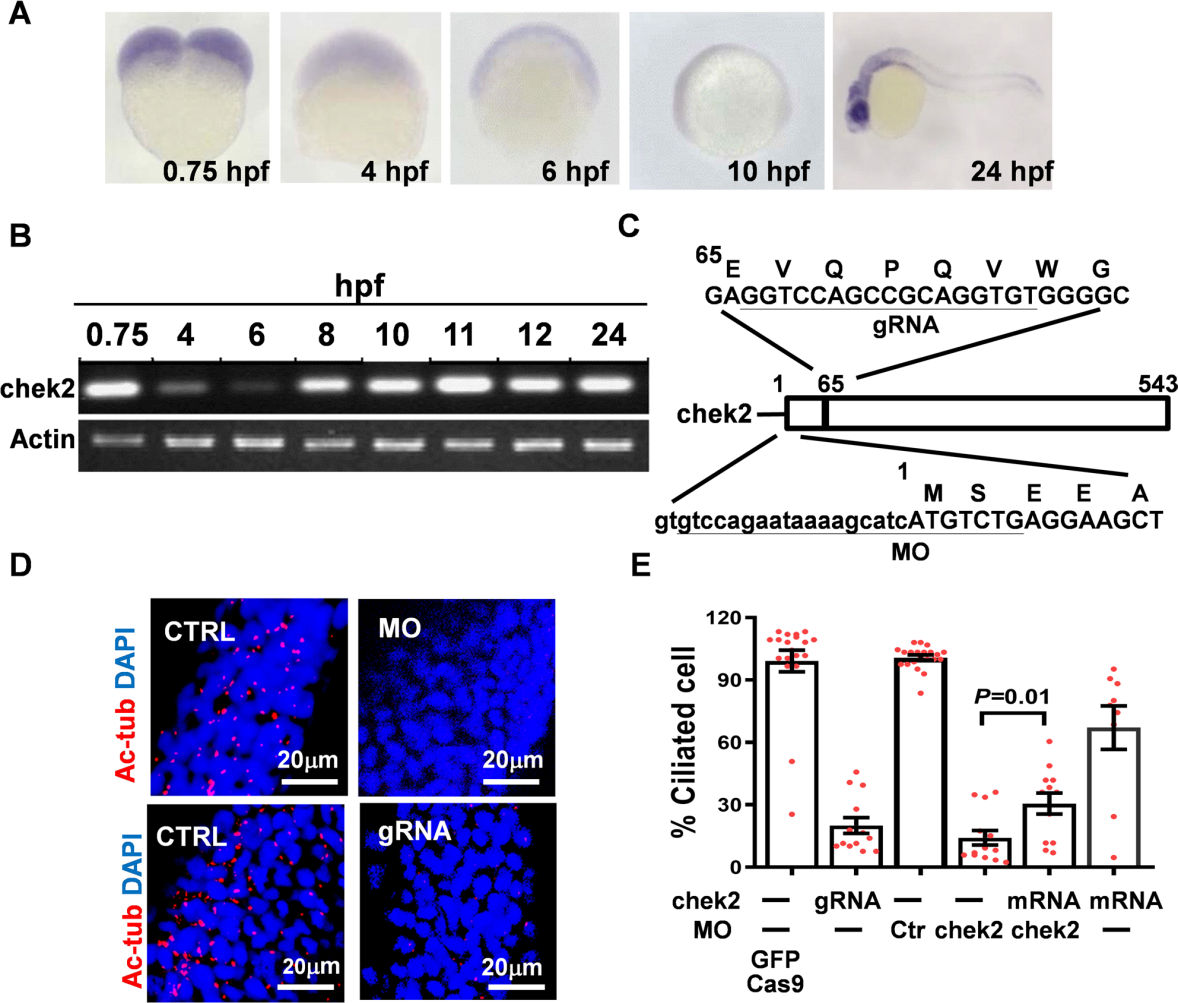
Zebrafish *chek2* is a maternal transcript required for primary ciliation during embryonic development. **A** Zebrafish chek2 transcript appears as a maternal transcript that later changed to zygotic expression at the anterior region determined by whole-mount in situ hybridization and (**B**) RT-PCR (upper row, chek2; lower row, actin). The hpf means hours post fertilization. All embryos are in lateral view, with the animal pole to the top (0.75 hpf), dorsal (4–10 hpf) to the right, and anterior (10 and 24 hpf) to the left. **C** The sgRNA and the morpholino (MO) binding sites in the chek2 cDNA locus. **D** Primary cilia stained with acetylated tubulin (Ac-tub) antibody in the head primordium (2500 µm2) at the bud stage. **E** Cells labeled with DAPI that contain primary cilia were calculated as the percentage. Embryo numbers in each injection from the left to the right were (GFP+Cas9 mRNA: 19, chek2 sgRNA+Cas9 mRNA:13, Control MO:21, chek2 MO: 13, chek2 MO+ human chek2 mRNA: 12, human chek2 mRNA: 9)

To further investigate the role of CHEK2 in vivo, we employed zebrafish as a model system. We first analyzed the expression pattern of zebrafish *chek2* during embryogenesis. Both in situ hybridization (Fig. 3A) and RT-PCR (Fig. 3B) detected *chek2* as a maternal transcript shortly after fertilization. These maternal transcripts gradually diminished over time, while zygotic expression emerged around 8–10 h post-fertilization (hpf) and became more prominent in the head region by 24 hpf.

The function of *chek2* was examined after depleting its mRNA using antisense morpholino or sgRNA/Cas9 mRNA (Fig. 3C). Primary cilia around the eye primordia at the lateral gastrula stage were visualized by acetylated tubulin staining, and they were significantly reduced upon *chek2* knockdown by either morpholino or gRNA (Fig. 3D). The proportion of ciliated cells was rescued by co-injection of human *CHEK2* mRNA (Fig. 3E), suggesting a conserved function across species. These findings demonstrate the role of *chek2* in primary cilia in vivo.

### CHEK2 regulates trophoblast cell invasion via primary cilia

**Fig. 4:**
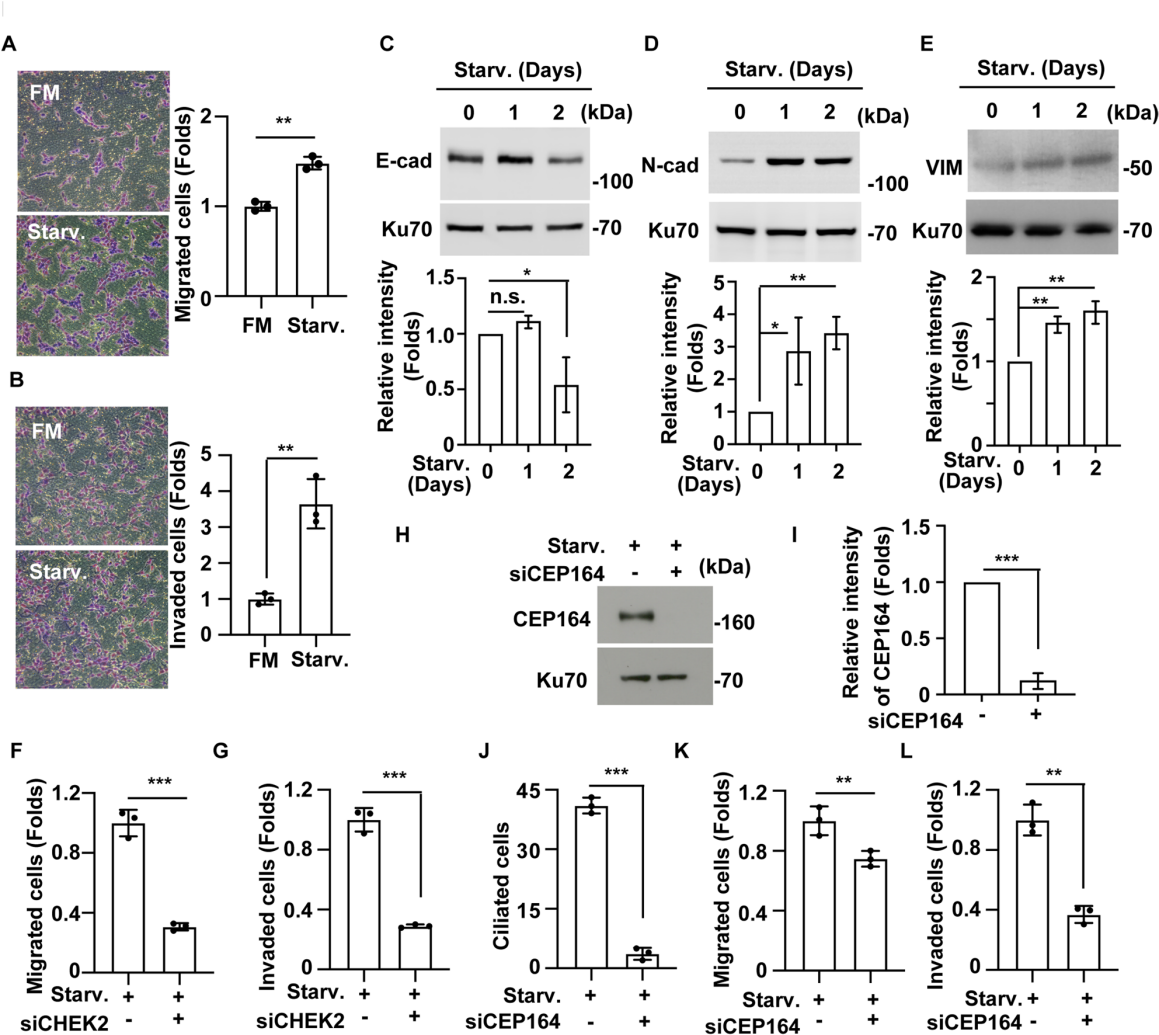
CHEK2 promotes trophoblast cell invasion upon serum deprivation. **A**-**B** Serum starvation promotes trophoblast HTR-8 cell migration and invasion. Left: visualization of cells that migrated to the lower chamber. Right: Quantitative results of (**A**) migrated or (**B**) invaded cells in full-medium (FM) or serum-starved medium (Starv.). **C**-**E** Western blotting showing that serum starvation reduced (**C**) E-cadherin (E-cad, epithelial marker), but promoted (**D**) N-cadherin (N-cad, mesenchymal marker) and (**E**) vimentin (VIM, mesenchymal marker). Ku70 and actin are gel-loading controls. **F**-**G** Depletion of CHEK2 by siCHEK2 inhibits trophoblast HTR-8 cell (**F**) migration and (**G**) invasion. **H**-**L** Depletion of CEP164 inhibited primary cilia, cell migration, and invasion. **H**-**I** Efficient depletion of CEP164 by siRNA. **H** Extracts of cells transfected with siCEP164 were analyzed by Western blotting with antibodies against CEP164 and Ku70. **I** Quantitative results for the relative intensity of CEP164 in **H**. Reduced numbers of (**J**) ciliated, (**K**) migrated, or (**L**) invaded HTR-8 cells in the presence of siCEP164 under serum starvation

Our data show that CHEK2 regulates primary cilia, which are known to facilitate trophoblast invasion during placenta formation at the beginning of pregnancy [25, 26]. Therefore, we examined whether CHEK2 regulates trophoblast cell invasion. Upon serum starvation, we observed increased trophoblast cell migration (Fig. 4A) and invasion (Fig. 4B). In addition, we detected a reduction in E-cadherin expression, along with an upregulation of N-cadherin and vimentin (Fig. 4C-E). These molecular changes are often associated with enhanced migratory capacity in trophoblast cells.

Next, we examined the role of CHEK2 in trophoblast cell migration and invasion. Depletion (Fig. 4F-G) or inhibition of CHEK2 (Supplementary Fig. S3A-B) reduced trophoblast cell migration (Fig. 4F and Supplementary Fig. S3A) and invasion (Fig. 4G and Supplementary Fig. S3B) under serum deprivation. We then checked whether primary cilia play a role here by depleting ciliary protein CEP164 using siRNA transfection (Fig. 4H-I); this resulted in a reduced proportion of ciliated cells (Fig. 4J), decreased trophoblast cell migration (Fig. 4K), and invasion (Fig. 4L). These data were confirmed when the other ciliary protein IFT88 was depleted (Supplementary Fig. S3C-F). To further confirm that CHEK2-mediated primary cilia contribute to trophoblast cell migration and invasion, we performed these experiments under full culture medium conditions, in which trophoblast cells do not form primary cilia. Neither inhibition nor depletion of CHEK2 affected trophoblast cell migration or invasion (Supplementary Fig. 3G-J), suggesting that CHEK2 regulates these processes through primary cilia. Collectively, our data demonstrate that CHEK2 promotes trophoblast cell migration and invasion via primary cilia.

### CHEK2 promotes pancreatic cancer cell migration and invasion upon glutamine deprivation

**Fig. 5:**
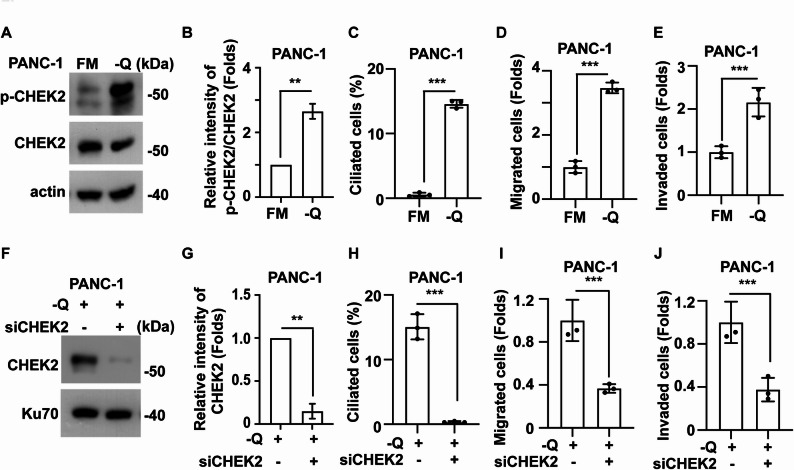
CHEK2 promotes PDAC cell invasion upon Gln deprivation. **A**-**E** Gln deprivation activates CHEK2 and promotes primary ciliation, migration, and invasion. **A** CHEK2 was phosphorylated in PANC-1 cells under Gln deprivation. Extracts of PANC-1 cells under full-medium (FM) or Gln deprivation (-Q) were analyzed by Western blotting with antibodies against phosphorylated CHEK2 (p-CHEK2), CHEK2, and actin. **B** Quantitative results for the relative intensity of p-CHEK2/CHEK2 in **A**. Quantitative results of (**C**) ciliated, (**D**) migrated, or (**E**) invaded cells in full-medium (FM) or Gln-deprived medium (-Q). **D**-**H** CHEK2 promotes PDAC invasion under glutamine deprivation. **F**-**J** Depletion of CHEK2 reduced primary cilia, migration, and invasion under Gln deprivation. **F** Extracts of PANC-1 cells in the absence or presence of siCHEK2 were analyzed by Western blotting with antibodies against CHEK2 and Ku70. **G** Quantitative results for the relative intensity of CHEK2 in **F**. **H**-**J** Quantitative results of (**H**) ciliate, (**I**) migrated, or (**J**) invaded cells in control or CHEK2-deficient (siCHEK2) cells under Gln deprivation (-Q). **p < 0.01, ***p < 0.001. These results are mean ± SD from three independent experiments

Our data demonstrated that CHEK2 maintained primary cilia for trophoblast invasion, which resembles cancer metastasis. Since pancreatic ductal adenocarcinoma (PDAC) increases its invasive ability when deprived of glutamine (Gln, Q) [37], we investigated whether Gln deprivation activates CHEK2. We deprived a human pancreatic ductal adenocarcinoma cell line PANC-1 of Gln, and found that CHEK2 was activated (Fig. 5A-B), primary cilia grew (Fig. 5C), PANC-1 cell migration and invasion were enhanced (Fig. 5D-E). Depletion of CHEK2 (Fig. 5F-G) reduced primary cilia (Fig. 5H), cell migration (Fig. 5I), and cell invasion (Fig. 5J). Treating PANC-1 cells with CHEK2 inhibitor also reduced PDAC ciliation, migration, and invasion (Supplementary Fig. S3K-M). These findings demonstrate that Gln deprivation activates CHEK2, which is required to induce primary cilia formation for cell migration and invasion in PDAC.

### CHEK2 maintains primary cilia stability

Next, we investigated the molecular mechanism by which CHEK2 regulates primary cilia. In the presence of CHEK2i, appendage components such as CEP164, CEP170, and ODF2 appeared comparable to those in control cells (Supplementary Fig. S4A-C). TTBK2 was still recruited to the mother centriole (Supplementary Fig. S5A-B) and CP110 was properly removed (Supplementary Fig. S5C-D), suggesting that CHEK2 does not affect the ciliogenesis machinery. Primary cilia were properly covered with ciliary vesicles (Supplementary Fig. S5E-F), indicating that CHEK2 does not participate in ciliary vesicle docking.

We examined cell cycles and found that serum deprivation led to G0/G1 arrest, but inhibition of CHEK2 did not disrupt G0/G1 arrest (Supplementary Fig. S6A-B), nor EdU incorporation (Supplementary Fig. S6C). These data suggest that CHEK2 does not affect serum deprivation-induced G0/G1 arrest.

**Fig. 6:**
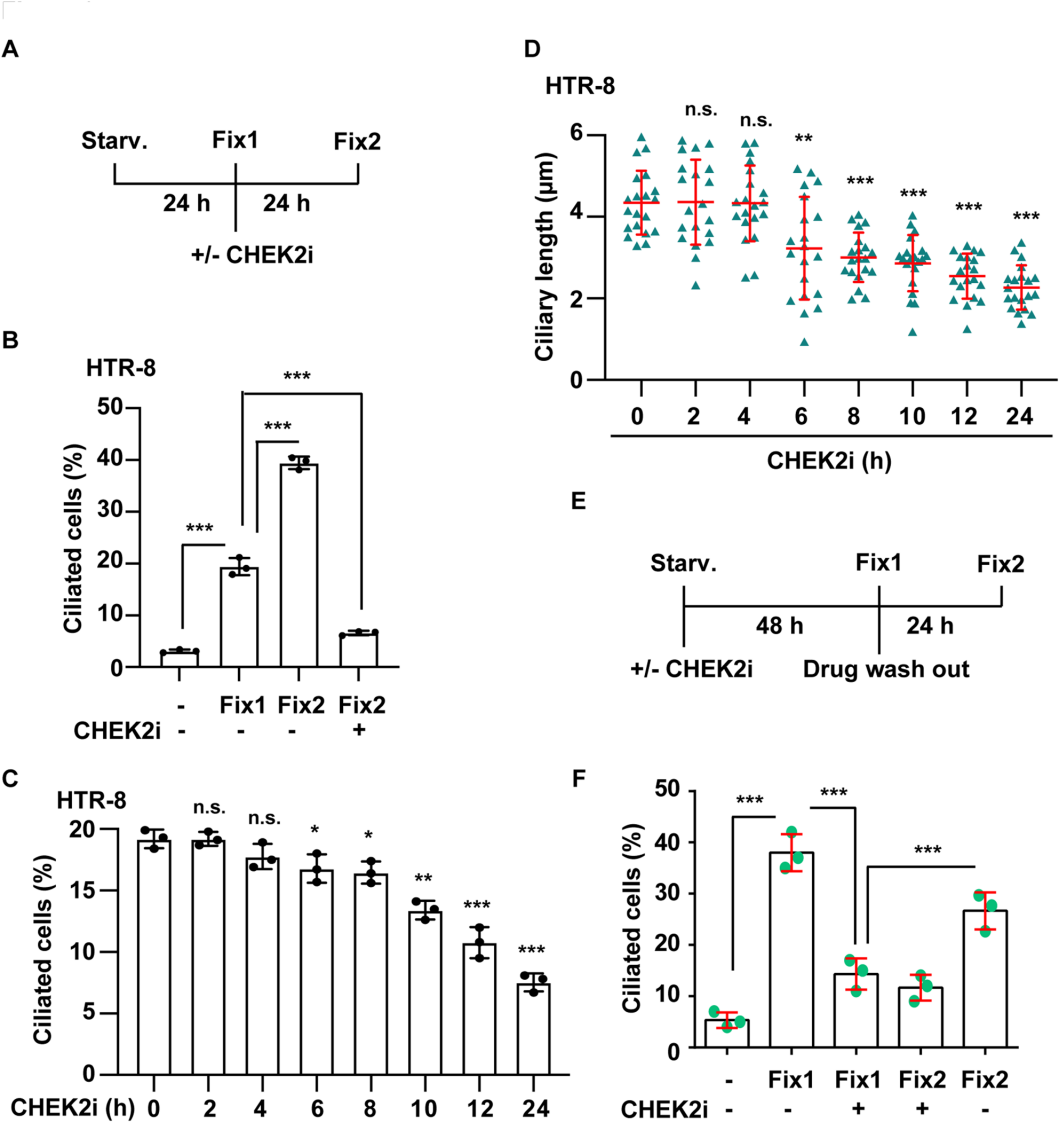
CHEK2 maintains the primary cilia of RPE1 cells. **A** Depiction of experimental scheme. Fix1: serum starvation for 24 h. Fix2: serum starvation for 48 h with or without 24 CHEK2 inhibition (+/-CHEK2i). **B** Quantitative results of ciliated cells. Time course of quantitation of (**C**) ciliated cells and (**D**) the length of primary cilia in HTR-8 cells starved for 24 h followed by treating CHEK2 inhibitor for 0, 2, 4, 6, 8, 10, 12, 24 h. (E-F) Regrowth of primary cilia was observed after CHEK2i washout. **E** Schematic of the experimental protocol. Fix 1: Cells were starved for 48 h with or without CHEK2i (+/− CHEK2i). Fix 2: After treatment with CHEK2i for 48 h, the drug was washed out and the cells were cultured in drug‑free medium for an additional 24 h. **F** Quantification of cells with primary cilia in **E**. n.s.: no significance, * p < 0.05, **: p < 0.01, ***:p < 0.001. These results are mean ± SD from three independent experiments

Next, we investigated whether active CHEK2 is required to maintain primary cilia. RPE1 cells were first starved for 24 h to induce primary cilia formation, followed by CHEK2 inhibition for an additional 24 h under starvation (Fig. 6A). Serum starvation for 24 h induced primary cilia formation, and a further 24 h of starvation induced more primary cilia (Fig. 6B). However, when CHEK2 was inhibited for an additional 24 h after starvation, the existing primary cilia were disassembled in both RPE-1 (Fig. 6B) and HTR-8 cells (Supplementary Figs. S6D). These data suggest that active CHEK2 maintains primary cilia during serum deprivation. We also followed the time course of ciliary disassembly at 2-h intervals. After 6 h of CHEK2 inhibitor treatment, both the proportion of ciliated cells and the length of primary cilia were gradually reduced (Fig. 6C-D). To further clarify the role of CHEK2 in primary cilia, we serum-starved HTR-8 cells for 48 h in the absence or presence of CHEK2i, followed by washing out the drug and culturing the cells for an additional 24 h (Fig. 6E). In the presence of CHEK2i, starvation-induced primary cilia were markedly reduced. However, upon removal of CHEK2i, primary cilia reappeared (Fig. 6F). Thus, CHEK2 maintains primary cilia during serum deprivation.

### CHEK2 promotes Aurora A degradation

**Fig. 7:**
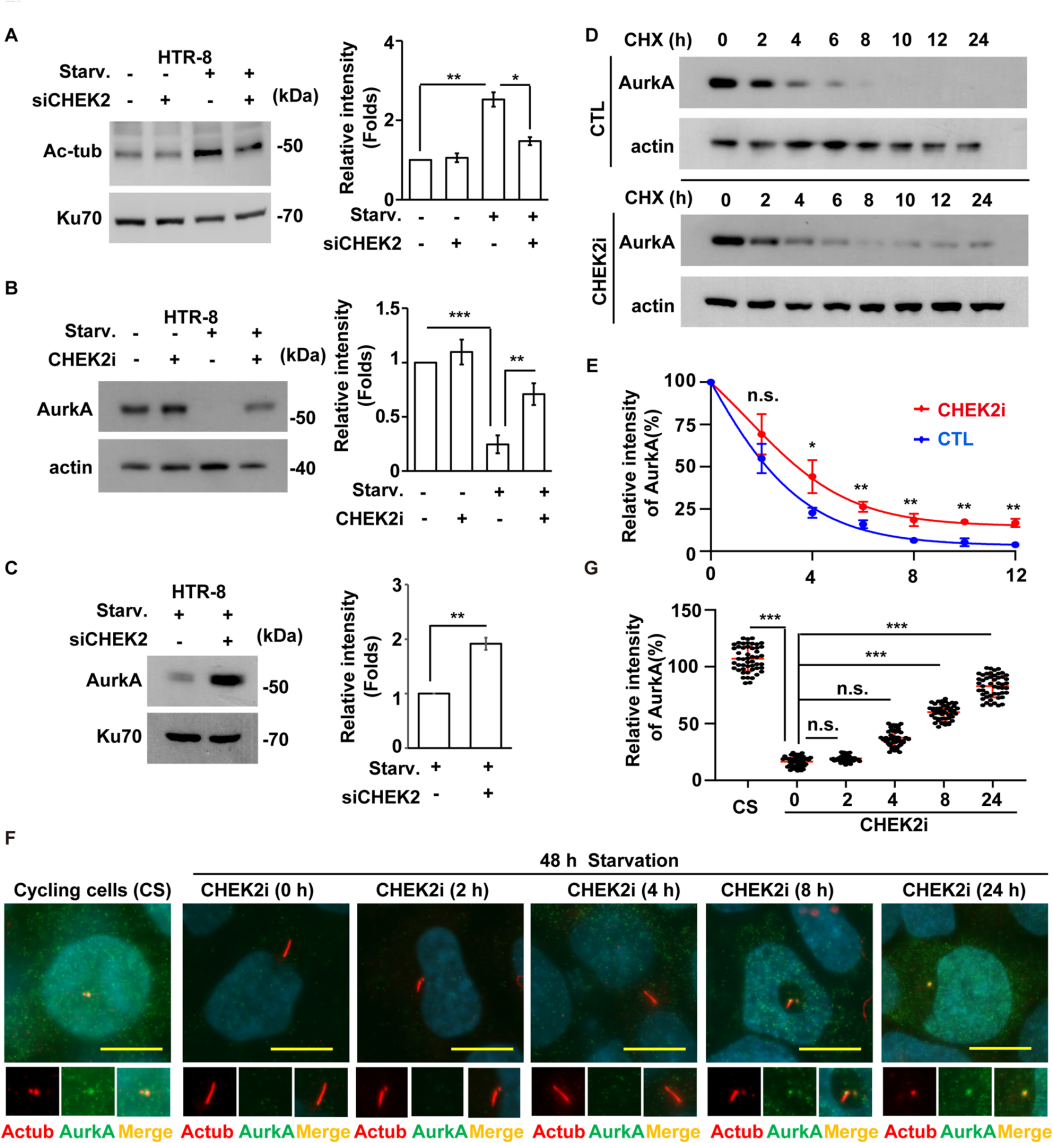
CHEK2 promotes AurkA degradation. **A** Depletion of CHEK2 reduced the amount of acetylated tubulin under serum starvation. Extracts of wild-type or CHEK2-depleted HTR-8 cells in the absence or presence of serum starvation (Starv.) were analyzed by Western blotting with antibodies against acetylated tubulin (Ac-tub), phosphorylated CHEK2 (p-CHEK2), and Ku70. **B**-**C** Inhibition or depletion of CHEK2 restores AurkA under serum deprivation. Extracts of HTR-8 cells treated with (**B**) CHEK2 inhibitor (CHEK2i) or (**C**) siRNA against CHEK2 (siCHEK2) were analyzed by Western blotting with antibodies against AurkA (AurA), Ku70, and actin. **D**-**E** Inhibition of CHEK2 increased AurkA protein stability. **D** Extracts of control (CTL) or CHEK2-inhibited (CHEK2i) cells with cycloheximide (CHX) treatment for 0, 2, 4, 6, 8, 10, 12, and 24 h were analyzed by Western blotting with antibodies against AurkA (AurA) and actin. **E** Quantitative results of **D**. **F**-**G** AurkA was reduced after 48 h of serum starvation and reappeared at the base of primary cilia. **F** AurkA in HTR-8 cells cultured in full medium (cycling cells) or serum-starved medium (48 h starvation) in the absence (CHEK2i 0 h) or presence of CHEK2i for different time periods (CHEK2i 2 h, 4 h, 8 h, and 24 h) was examined by immunofluorescence staining with antibodies against AurkA and acetylated tubulin (Actub). DNA were stained with DAPI. Scale bar: 10 μm. **G** Quantitative results for the relative intensity of AurkA in **F**. n.s.: no significance, *p < 0.05, **p < 0.01. These results are mean ± SD from three independent experiments

Acetylation of K40 in α-tubulin affects microtubule stability and function [38], while the axoneme is highly acetylated [36]. The disassembly of primary cilia is regulated by Aurora A, which causes tubulin deacetylation to destabilize ciliary axoneme [20, 22, 24]. We therefore examined tubulin acetylation and Aurora kinase A (AurkA). Serum deprivation increased the amount of acetylated tubulin; while depletion or inhibition of CHEK2 reduced it in HTR-8 cells (Fig. 7A), RPE-1 cells (Supplementary Fig. S7A), and NIH-3T3 cells (Supplementary Fig. S7B). The data suggest that CHEK2 maintains tubulin acetylation.

Next, the expression of AurkA was examined. Upon serum deprivation, AurkA levels were reduced (Fig. 7B); however, this reduction was reversed when CHEK2 was inhibited or depleted (Fig. 7B-C). Furthermore, we found that, in PANC-1 cells, Gln deprivation reduced AurkA protein levels, and this effect was reversed by CHEK2 depletion (Supplementary Fig. S7C), suggesting that CHEK2 reduced AurkA expression in both trophoblast and PDAC cells. Next, we evaluated AurkA stability in HTR-8 cells. Protein translation was blocked by cycloheximide (CHX), and the levels of AurkA were assessed by Western blotting. In control cells, AurkA was barely detectable 8 h after CHX treatment; but remained detectable 24 h after CHX treatment when CHEK2 was inhibited (Fig. 7D-E). These results indicate that CHEK2 facilitates AurkA degradation.

The subcellular localization of AurkA was examined. In cycling cells, AurkA was observed at the centriole (Fig. 7F). After serum deprivation, centriolar AurkA disappeared and primary cilia were formed. However, when CHEK2 was inhibited in serum-starved cells, centriolar AurkA gradually increased and the primary cilia progressively decreased (Fig. 7F-G). Thus, CHEK2 inhibition increases centriolar AurkA and promotes cilia disassembly.

In addition to AurkA, the acetyltransferase α-tubulin acetyltransferase 1 (ATAT1) and the deacetylases histone deacetylase 6 (HDAC6) and Sirtuin 2 (SIRT2) were also examined. Inhibition of CHEK2 in trophoblast cells did not affect the expression of ATAT1, SIRT2, or total HDAC6, but it increased the phosphorylated form of HDAC6 (Supplementary Fig. 7D–E). Thus, both AurkA and HDAC6 phosphorylation are regulated by CHEK2 activation.

### CHEK2 maintains primary cilia by regulating autophagy

Serum deprivation activates autophagy, which often interacts with primary cilia [14]. In HTR-8 cells, serum deprivation increased LC3 puncta formation and the LC3-II/LC3-I ratio (Supplementary Fig. S8A-B); and depletion of autophagy effector, ATG7, reduced primary cilia and invasive ability (Supplementary Fig. S8C-E). Thus, in HTR-8 cells, starvation also activates autophagy to maintain primary cilia and to promote cell invasion.

**Fig. 8:**
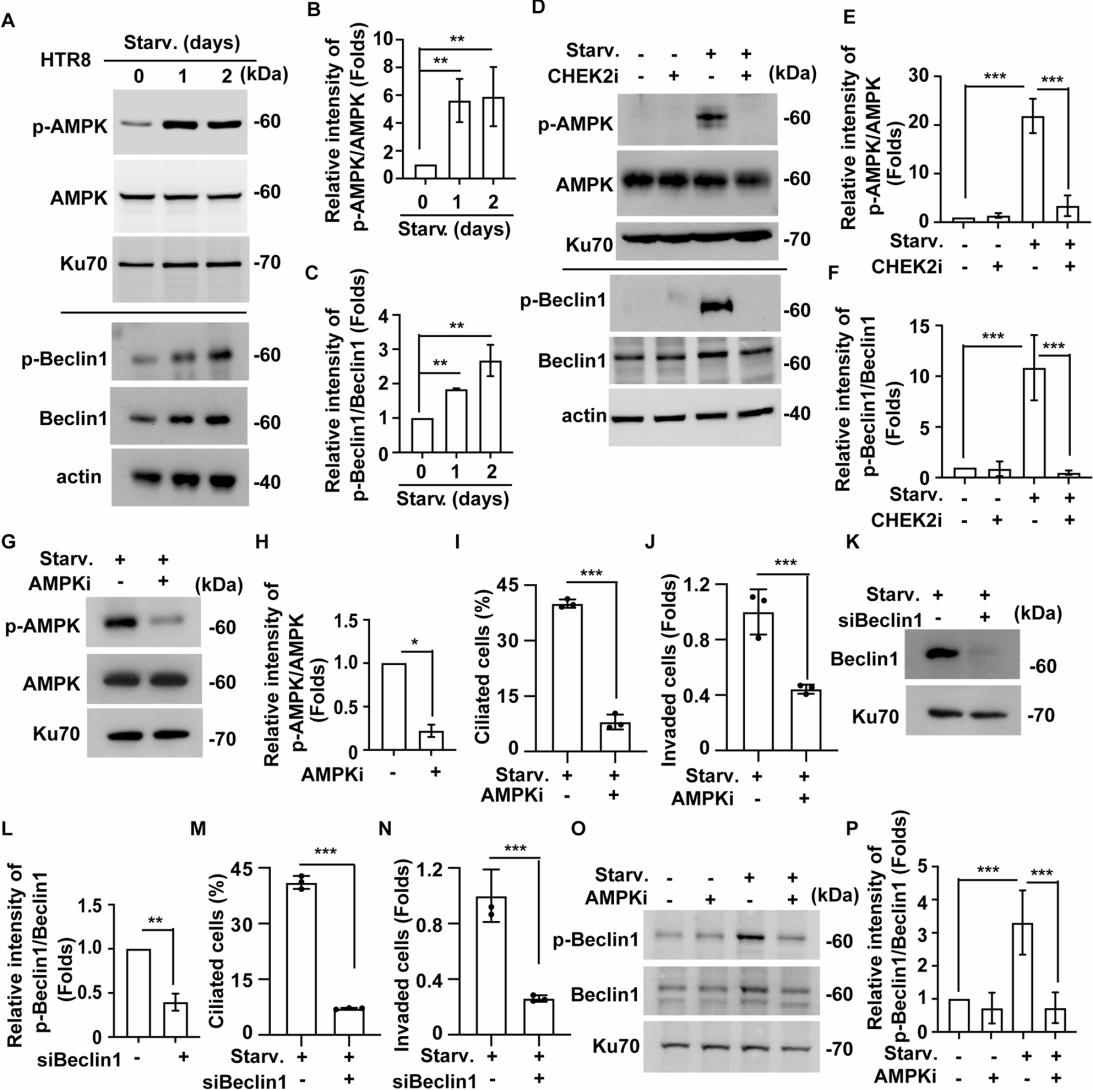
CHEK2 activates autophagy to promote primary ciliation and cell invasion under serum starvation. **A**-**C** AMPK and Beclin 1 were activated during serum starvation. Extracts of HTR-8 cells serum-starved for 1 or 2 days were analyzed by Western blotting with antibodies against phosphorylated AMPK (p-AMPK), AMPK, phosphorylated ULK1, ULK1, phosphorylated Beclin1 (p-Beclin1), Beclin1, Ku70, and actin. **B**-**C** Quantitative results for the relative intensity of (**B**) p-AMPK/AMPK and (**C**) p-Beclin1/Beclin1 in **A**. **D**-**F **Inhibition of CHEK2 alleviated AMPK and Beclin1 activation. Extracts of HTR-8 cells in the absence or presence of CHEK2 inhibitor (CHEK2i) with or without serum starvation were analyzed by Western blotting with antibodies against phosphorylated AMPK (p-AMPK), AMPK, phosphorylated Beclin1 (p-Beclin1), Beclin1, Ku70, and actin. **E**-**F** Quantitative results for the relative intensity of (**E**) p-AMPK/AMPK and (**F**) p-Beclin1/Beclin1 in **D**. **G**-**J** Inhibition of AMPK reduced primary cilia and trophoblast cell invasion. **G** AMPK was inhibited by treating cells with AMPK inhibitor. Extracts of serum-starved HTR-8 cells in the presence or absence of AMPK inhibitor (AMPKi) were analyzed by Western blotting with antibodies against phosphorylated AMPK (p-AMPK), AMPK, and Ku70. **H** Quantitative results for the relative intensity of p-AMPK/AMPK in **G**. **I**-**J** Inhibition of AMPK reduced primary cilia and cell invasion HTR-8 cells. Quantitative results of (**I**) ciliated and (**J**) invaded cells in the absence or presence of AMPKi. **K**-**N** Depletion of Beclin1 reduced primary cilia and invasion of trophoblast cells. **K**-**L** Beclin1 was depleted efficiently. **K** Extracts of HTR-8 cells transfected with siRNA against Beclin1 were analyzed by Western blotting with antibodies against Beclin1 and actin. **L** Quantitative results for the relative intensity of p-Beclin1/Beclin1 in (K). (M-N) Quantitative results of (**M**) ciliated and (**N**) invaded cells of wild-type or Beclin1-depleted (siBeclin1) cells. **O**-**P** Inhibition of AMPK alleviated Beclin1 activation. **O** Extracts of cells treated with or without AMPK inhibitor (AMPKi) were analyzed by Western blotting with antibodies against phosphorylated Beclin1 (p-Beclin1), Beclin1, and Ku70. **P** Quantitative results for the relative intensity of p-Beclin1/Beclin1 in **O**. ***p < 0.001. These results are mean ± SD from three independent experiments

Since CHEK2 is required for primary cilia, we examined whether CHEK2 activated autophagy. We assessed essential factors for autophagy initiation and nucleation, AMPK, ULK1, and Beclin1 [15, 39]. During serum deprivation, phosphorylation levels of AMPK and Beclin1 (Fig. 8A-C), but not ULK1 (Supplementary Fig. S8F), were increased in a time-dependent manner. When cells were treated with CHEK2i, phosphorylation levels of AMPK and Beclin1 were markedly reduced (Fig. 8D-F), suggesting that CHEK2 activity is required for proper activation of the autophagy pathway under serum starvation.

We treated HTR-8 cells with AMPK inhibitor (AMPKi), which reduced AMPK phosphorylation (Fig. 8G-H), and the proportion of ciliated cells (Fig. 8I) and invaded cells was decreased (Fig. 8J). Similarly, the depletion of Beclin1 led to reduced cell ciliation and invasion (Fig. 8K-N). Inhibition of AMPK further reduced Beclin1 phosphorylation (Fig. 8O-P), indicating that AMPK is upstream of Beclin1 activation. These data indicate that activation of the AMPK-Beclin1 autophagy pathway contributes to primary cilia maintenance during serum starvation.

**Fig. 9:**
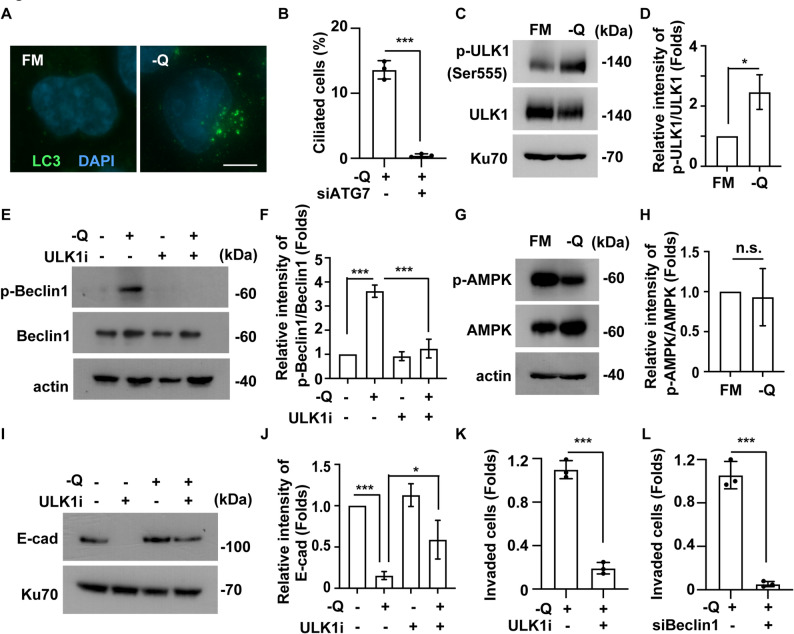
Glutamine deprivation enhances autophagy to promote primary ciliation and cell invasion. **A** Autophagy is activated by Gln deprivation. Autophagy was observed by immunofluorescence staining with antibodies against LC3 in PANC-1 cells cultured in full-medium (FM) or Gln-deprived medium (-Q). DNA were stained with DAPI. Scale bar: 10 μm. **B** Depletion of ATG7 inhibited primary cilia under Gln deprivation in PANC-1 cells. Quantitative results of ciliated cells in the absence or presence of siRNA against *ATG7*. **C-D** Gln deprivation activated ULK1 in PANC-1 cells. **C** Extracts of PANC-1 cells cultured in full-medium (FM) or Gln-deprived (-Q) medium were analyzed by Western blotting with antibodies against phosphorylated ULK1 (p-ULK1), ULK1, phosphorylated AMPK (p-AMPK), AMPK, Ku70, and actin. **D** Quantitative results for the relative intensity of p-ULK1/ULK1 in **C**. **E-F** Inhibition of ULK1 alleviated Beclin1 phosphorylation under Gln deprivation. (**E**) Extracts of PANC-1 cells were analyzed by Western blotting with antibodies against phosphorylated Beclin1 (p-Beclin1), Beclin1, E-cadherin (E-cad), Ku70, and actin. **F** Quantitative results for the relative intensity of p-Beclin1/Beclin1 in **E**. (**G-H**) AMPK was not activated during Gln deprivation in PANC-1 cells. **G** Extracts of PANC-1 cells cultured in full-medium (FM) or Gln-deprived (-Q) medium were analyzed by Western blotting with antibodies against phosphorylated AMPK (p-AMPK), AMPK, and Ku70. **H** Quantitative results for the relative intensity of p-AMPK/AMPK in **G**. (**I-J**) Inhibition of ULK1 alleviated EMT under Gln deprivation. **I** Extracts of PANC-1 cells were analyzed by Western blotting with antibodies against E-cadherin (E-cad) and Ku70. **J** Quantitative results for the relative intensity of E-cad in **I**. **K-L** Inhibition of ULK1 or depletion of Beclin1 alleviated PANC-1 cell invasion under Gln deprivation. Quantitative results of invaded cells in the absence or presence of (K) ULK1 inhibitor (ULKi) or (**L**) siRNA against *Beclin1* (siBeclin1). ***:*p* < 0.001. These results are mean ± SD from three independent experiments

In PANC-1 cells, Gln deprivation increased LC3 puncta formation (Fig. 9A), and depletion of ATG7 resulted in a reduction of ciliated cells (Fig. 9B). Thus, Gln deprivation of PANC-1 cells also increased autophagy to support primary cilia. Gln deprivation activated ULK1 (Fig. 9C-D) and Beclin1 (Fig. 9E-F), but not AMPK (Fig. 9G-H). Inhibition of ULK1 reduced E-Cadherin (Fig. 9I-J) and attenuated Gln deprivation-induced cell invasion (Fig. 9K). Similarly, Beclin1 depletion reduced PANC-1 cell invasion when deprived of Gln (Fig. 9L). Thus, our data suggest that autophagy enhances primary ciliation for cell invasion upon metabolic stresses.

Previous studies have shown that AurkA functions as a negative regulator of autophagy in breast cancer cells. Inhibition of AurkA induces autophagy through suppression of mTOR signaling, whereas its overexpression suppresses autophagic activity [40]. Similarly, AurkA activation stabilizes oncogenic proteins such as YAP by preventing their autophagy-mediated degradation [41]. These findings suggest that AurkA acts upstream of the autophagy-lysosomal system. However, it remains unclear whether AurkA regulates autophagy in trophoblast cells. To investigate the interplay between AurkA and autophagy, we first examined whether AurkA inhibition affects autophagic activity. HTR-8 cells were treated with the selective AurkA inhibitor (Alisertib, 10 µM), and analysis of the LC3-II/LC3-I ratio revealed no significant change upon AurkA inhibition, indicating that AurkA probably does not actively regulate autophagy in trophoblast cells (Supplementary Fig. S9A). Next, we tested whether autophagy influences AurkA levels. When autophagy was inhibited by bafilomycin A1 treatment and LC3 II to I ratio increased significantly, phosphorylated and total AurkA protein levels were not much changed (Supplementary Fig. S9B). Thus, autophagy and AurkA appear independently regulated by CHEK2 in trophoblast cells.

## Discussion

In this article, we demonstrate that CHEK2 plays a critical role in maintaining primary cilia to promote trophoblast and pancreatic cancer cell invasion under metabolic stress conditions. CHEK2 promotes primary cilia by activating autophagy and preventing ciliary disassembly. Under serum deprivation, CHEK2 induces autophagy through activation of the AMPK-Beclin 1 axis, whereas under Gln deprivation, it activates the ULK1-Beclin 1 axis. These findings suggest that CHEK2 triggers distinct autophagy pathways in response to different metabolic stresses to support cilia. In addition to promoting autophagy, CHEK2 also facilitates the degradation of AurkA, thereby maintaining axoneme stability. Inhibition of autophagy did not alter AurkA protein levels, suggesting that the CHEK2-AurkA axis and CHEK2-autophagy pathway function independently to regulate primary cilia. Taken together, our study uncovers a novel role for CHEK2 in regulating primary cilia to promote cell invasion during metabolic stress.

Our manuscript proposes that CHEK2 regulates primary cilia at both the formation and stabilization stages. Specifically, CHEK2 activates autophagy, which has been reported to promote ciliogenesis, as disruption of autophagy results in reduced cilia formation. In addition, CHEK2 contributes to cilia stability by suppressing AurkA accumulation, a well-established driver of cilia disassembly. Thus, under metabolic stress, we hypothesize that CHEK2 activates autophagy to facilitate cilia formation, while simultaneously inhibiting AurkA to prevent cilia resorption, thereby ensuring the stabilization of primary cilia. A protein that plays a dual role in both promoting ciliogenesis and maintaining cilia stability has been reported previously. For example, TTBK2, a kinase known to initiate primary cilia growth, also stabilizes cilia by preventing their disassembly [21]. Therefore, CHEK2 may function in a similar manner under metabolic stress.

Only approximately 15% of PANC-1 cells grew primary cilia under glutamine-deprived conditions; however, our data demonstrate that inhibition of ciliogenesis resulted in a marked reduction (more than 50%) of cell migration and invasion. This finding suggests that primary cilia influence the migratory and invasive behavior of the cell population as a whole. In line with the concept that rare subpopulations can disproportionately orchestrate collective invasion, a recent study shows that scarce leader cells can strongly direct tumor progression and invasion by guiding the behavior of follower cells [42]. We thus propose that the ciliated cells in our study may belong to a specialized subpopulation that acts as leader-like cells, which are widely recognized as drivers of collective invasion through their ability to sense and transduce microenvironmental signals to surrounding non-ciliated cells. Nevertheless, this interpretation remains hypothetical and will require dedicated future studies to be formally validated.

The recent study shows that AurkA activation promotes cell‑cycle re‑entry from quiescence by driving primary cilium resorption under permissive, mitogen‑rich conditions [43]. In contrast, our experiments were performed under sustained metabolic stress, such as serum or glutamine deprivation, where mitogenic signaling is strongly suppressed and cells remain predominantly in G0/G1 despite the loss of primary cilia. Under these starvation conditions, CHEK2‑dependent AurkA degradation is required to maintain ciliary axonemes and to support invasion, but pharmacological inhibition of CHEK2, although leading to AurkA accumulation and cilia loss, does not increase the S‑phase population in FACS profiles and the EdU assay. One explanation is that AurkA activation alone is insufficient to overcome the strong anti‑proliferative signals imposed by nutrient deprivation, so that the CHEK2–AurkA axis primarily impacts ciliary stability and invasive behavior rather than cell‑cycle re‑entry in our study. These findings suggest that the outcome of AurkA activation, such as cell‑cycle re‑entry and ciliary disassembly, is highly context‑dependent, being permissive in growth factor-related conditions but constrained under metabolic stress.

A previous study shows that CHEK2 directly phosphorylates Beclin 1 to induce autophagy [44]. Here we observed that CHEK2 selectively activated different components of the autophagy machinery depending on the type of metabolic stress and the cell type. Specifically, in trophoblast cells under serum deprivation, CHEK2 activation leads to AMPK activation, whereas in PDAC cells under Gln deprivation, CHEK2 promotes ULK1 activation. This divergence may reflect the distinct metabolic wiring and stress adaptation mechanisms in different cell types. Trophoblasts, which are highly sensitive to growth factors and nutrient availability during placental development, may rely more heavily on the AMPK axis as a primary energy sensor under serum-starved conditions [45]. Activation of AMPK in this context could help restore energy balance and promote cell survival through autophagy. AMPK activation in trophoblasts is also important to maintain cilia for trophoblast invasion during normal placenta development.

In contrast to trophoblasts, PDAC cells often exhibit high Gln dependence for proliferation and survival due to rewired metabolic pathways [37, 46]. When deleted of Gln, ULK1 activation by CHEK2 may represent a more direct mechanism to induce autophagy. This suggests that in PDAC cells, CHEK2 may signal directly to ULK1 bypassing AMPK to ensure a rapid autophagic response under conditions of metabolic vulnerability. The differential activation of AMPK or ULK1 by CHEK2 also raises the possibility that CHEK2 forms distinct protein complexes or undergoes context-specific post-translational modifications depending on the type of stress and cell type. Further studies are required to delineate the molecular determinants of this selectivity.

Our findings demonstrate that CHEK2 regulates cell invasion by preserving the structure and function of primary cilia, particularly under conditions of metabolic stress. Primary cilia act as signaling hubs for several oncogenic pathways, including Hedgehog, PDGFRα, and Wnt, all of which modulate cell polarity and motility [47–49]. When deprived of nutrients, cells often adapt to stress by remodeling signaling networks; here we show that CHEK2 maintains primary cilia through autophagy activation and stabilization of axonemal structures. By ensuring ciliary integrity during metabolic stress, CHEK2 may enhance the ability of cells to respond to micro-environmental cues that promote invasion. The cilia-mediated invasion pathway may be particularly relevant in the context of cancer cells, such as in PDAC, that are exposed to tumor microenvironment when deprived of Gln. Our data thus highlight an unexpected pro-invasive role of CHEK2 that complements its function in centrosome homeostasis, suggesting a more complex and context-dependent role of CHEK2 in tumor progression and metastasis.

## Conclusions

In this study, we identified a novel function of CHEK2 in regulating primary cilia, which in turn promotes trophoblast and pancreatic cancer cell invasion. Mechanistically, CHEK2 activates autophagy and facilitates the degradation of AurkA to preserve primary cilia integrity. Together, these findings reveal a previously unrecognized and versatile role of CHEK2 as a metabolic stress sensor that mediates primary cilia to ensure proper cell invasion.

## Supplementary Information

Supplementary Material 1.

Supplementary Material 2.

## Data Availability

No datasets were generated or analysed during the current study.

## Abbreviations

CHEK2: Checkpoint kinase 2
TTBK2: Tau tubulin kinase-2
IFT: Intraflagellar transport
AurkA: Aurora kinase A
HTR8: Human immortalized trophoblast HTR-8/SVneo cells
RPMI: Roswell Park Memorial Institute (RPMI)
RPE1: Human immortalized retinal pigment epithelial cells
DMEM: Dulbecco’s Modified Eagle Medium
FBS: Fetal bovine serum
MO: Morpholino
CHEK2i: CHEK2 inhibitor II
AMPKi: AMPK inhibitor
CHX: Cycloheximide
SDS-PAGE: SDS-polyacrylamide gel electrophoresis
PVDF: Polyvinylidene difluoride
HRP: Horseradish peroxidase
ECL: Enhanced chemiluminescence
KD-CHEK2: Kinase-dead CHEK2
FLAG-CHEK2: FLAG-tagged CHEK2
EMT: Epithelial-mesenchymal transition
PDAC: Pancreatic ductal adenocarcinoma
