# Supplementary material for: Checkpoint kinase 2 coordinates autophagy activation and Aurora kinase A degradation to regulate primary cilia for cell invasion
Source: Cell Commun Signal. 2026 May 22;24:401. doi: 10.1186/s12964-026-02953-6 (PMC13371562; doi:10.1186/s12964-026-02953-6)
Supplement: Supplementary file 1 — Supplementary Material 1. [file 12964_2026_2953_MOESM1_ESM.docx]

**Supplementary Figures**

Supplementary Figure S1


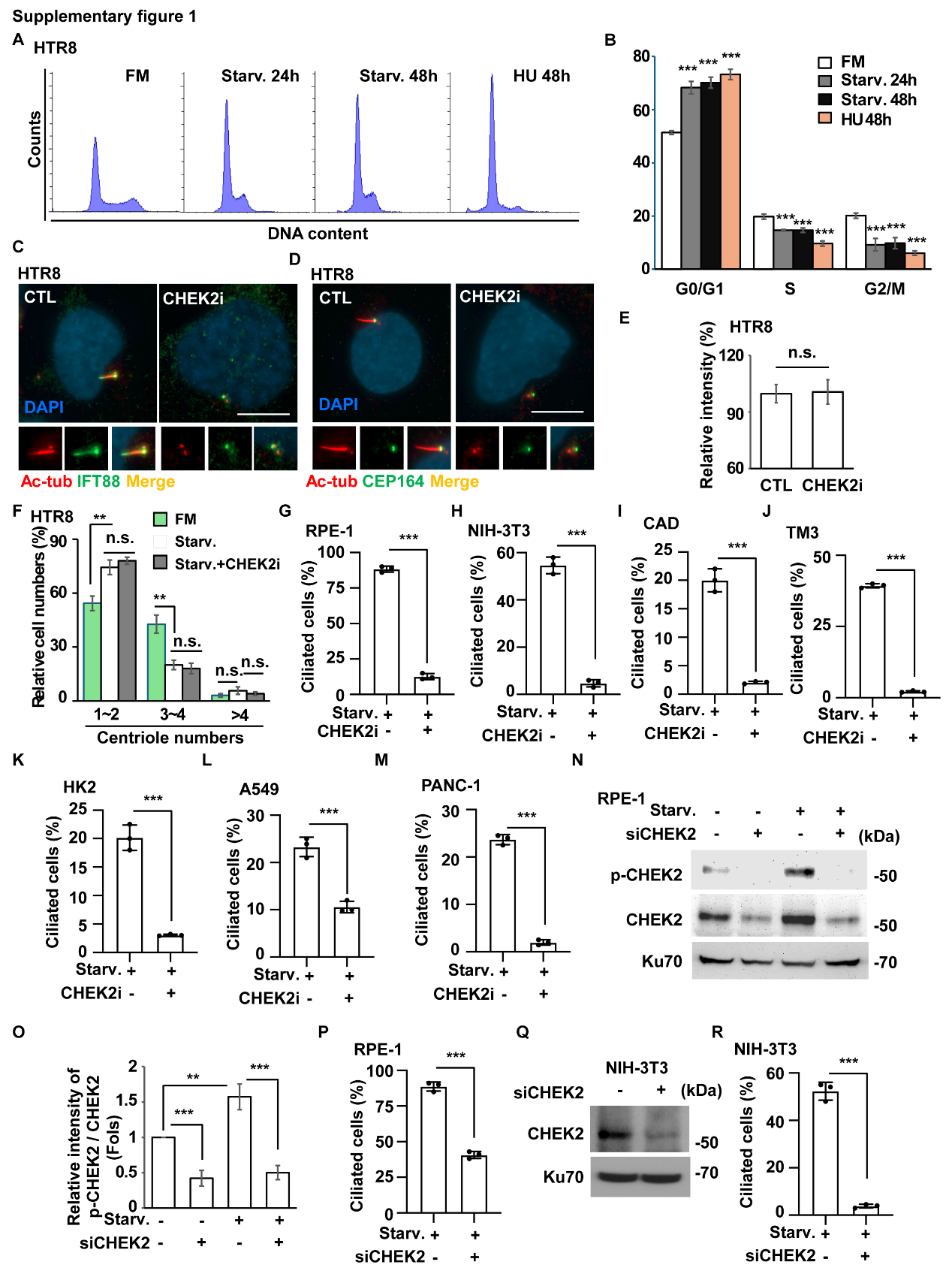


**Supplementary Figure S1. CHEK2 regulates primary cilia but not microtubule assembly.**

(**A-B**) Serum starvation induced G0/G1 arrest. Cell cycle profiles of HTR-8 cells were cultured in full medium (FM) or serum-starved medium for 24h (Starv. 24h) and 48h (Starv. 48h) or treated with hydroxyurea (HU). (B) Quantitative results of (A). (**C**) The primary cilia were reduced in CHEK2-inhibited HTR-8 cells under serum starvation. The axoneme (acetylated tubulin, Ac-tub) and intraflagellar transporter (IFT88) were observed by immunofluorescence staining. DNA were stained with DAPI. Scale bars: 10 μm. (**D-E**) Inhibition of CHEK2 did not affect centriolar appendages. (D) The axoneme (acetylated tubulin, Ac-tub) and distal appendages (CEP164) were observed by immunofluorescence staining. DNA were stained with DAPI. Scale bars: 10 μm. (E) Quantitative results of the relative intensity of CEP164 in (D). (**F**) Inhibition of CHEK2 did not affect the centriole copy numbers. Quantitative results of cells with 1-2 (non-duplicated), 3-4 (duplicated), or more than four (over-duplicated) centrioles in HTR8 cells cultured in full medium (FM) or serum-starved medium in the presence or absence of CHEK2i.(**G-M**) Inhibition of CHEK2 reduced primary cilia. Quantification of ciliated cells in the absence or presence of CHEK2 inhibitor (CHEK2i) in (G) RPE-1, (H) NIH-3T3, (I) CAD, (J) TM3, (K) HK2, (L) A549, and (M) PANC-1 cell lines. (**N-R**) Depletion of CHEK2 reduced primary cilia. (N and P) CHEK2 was depleted efficiently. Extracts of (N) RPE-1 and (Q) NIH-3T3 cells transfected with siRNA against the scramble control (CTL) or *CHEK2* were analyzed by Western blotting with antibodies against phosphorylated CHEK2 (p-CHEK2), CHEK2, Ku70, and tubulin. (O) Quantitative results for the relative intensity of p-CHEK2/CHEK2 are shown in (N). (P and R) Quantitative results of ciliated cells in CHEK2-deficient (P) RPE-1 or (R) NIH-3T3 cell lines. **p<0.01, ***p<0.001, n.s.: no significance. These results are mean ± SD from three independent experiments.

Supplementary Figure S2


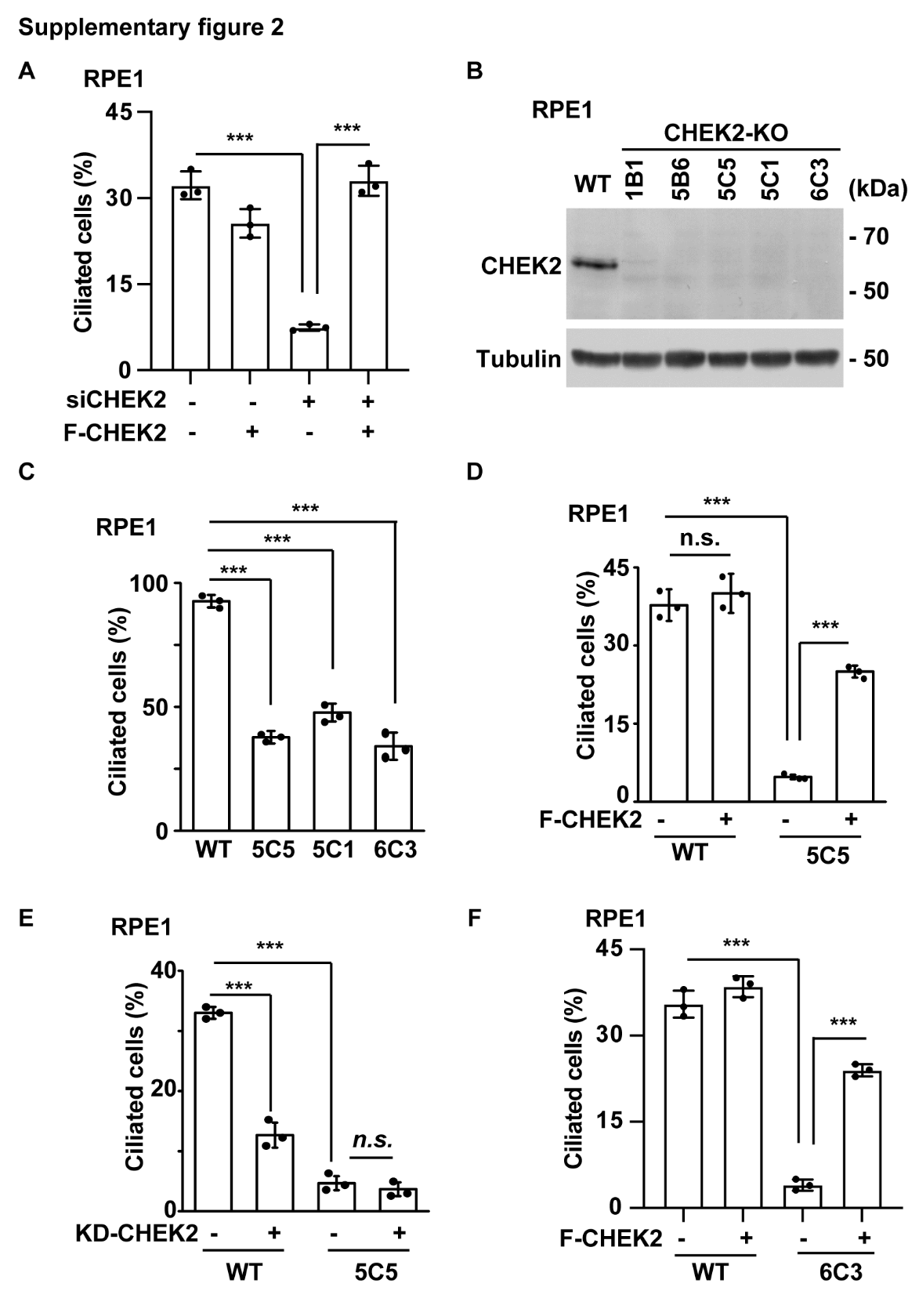


**Supplementary Figure S2. Overexpression of CHEK2 rescues primary cilia in CHEK2-deficient RPE-1 cells.**

(**A**) Overexpression of CHEK2 rescued the primary cilia. (E) Quantitative results of ciliated cells in RPE-1 cells transfected with or without siRNA against *CHEK2* (siCHEK2) in the absence or presence of FLAG-tagged CHEK2 (F-CHEK2). (**B-C**) Knockout of CHEK2 inhibited primary cilia in RPE-1 cells. (B) Extracts of wild-type (ET) or five different CHEK2 knockout (CHEK2-KO: 1B1, 5B6, 5C5, 5C1, and 6C3) RPE-1 cells were analyzed with antibodies against CHEK2 and tubulin. (C) Quantitative results of ciliated cells in wild-type (WT), 5C5, 5C1, or 6C3 cells. (**D-F**) Overexpression of wild-type, but not kinase-dead, CHEK2 rescued the defect of primary cilia. Quantitative results of ciliated cells in wild-type (WT), 5C5, or 6C3 CHEK2-KO RPE-1 cells with (D and F) overexpressing wild-type FLAG-tagged CHEK2 (F-CHEK2) or (E) kinase-dead CHEK2 (KD-CHEK2). n.s.: no significance, ***p<0.001. These results are mean ± SD from three independent experiments.

Supplementary Figure S3


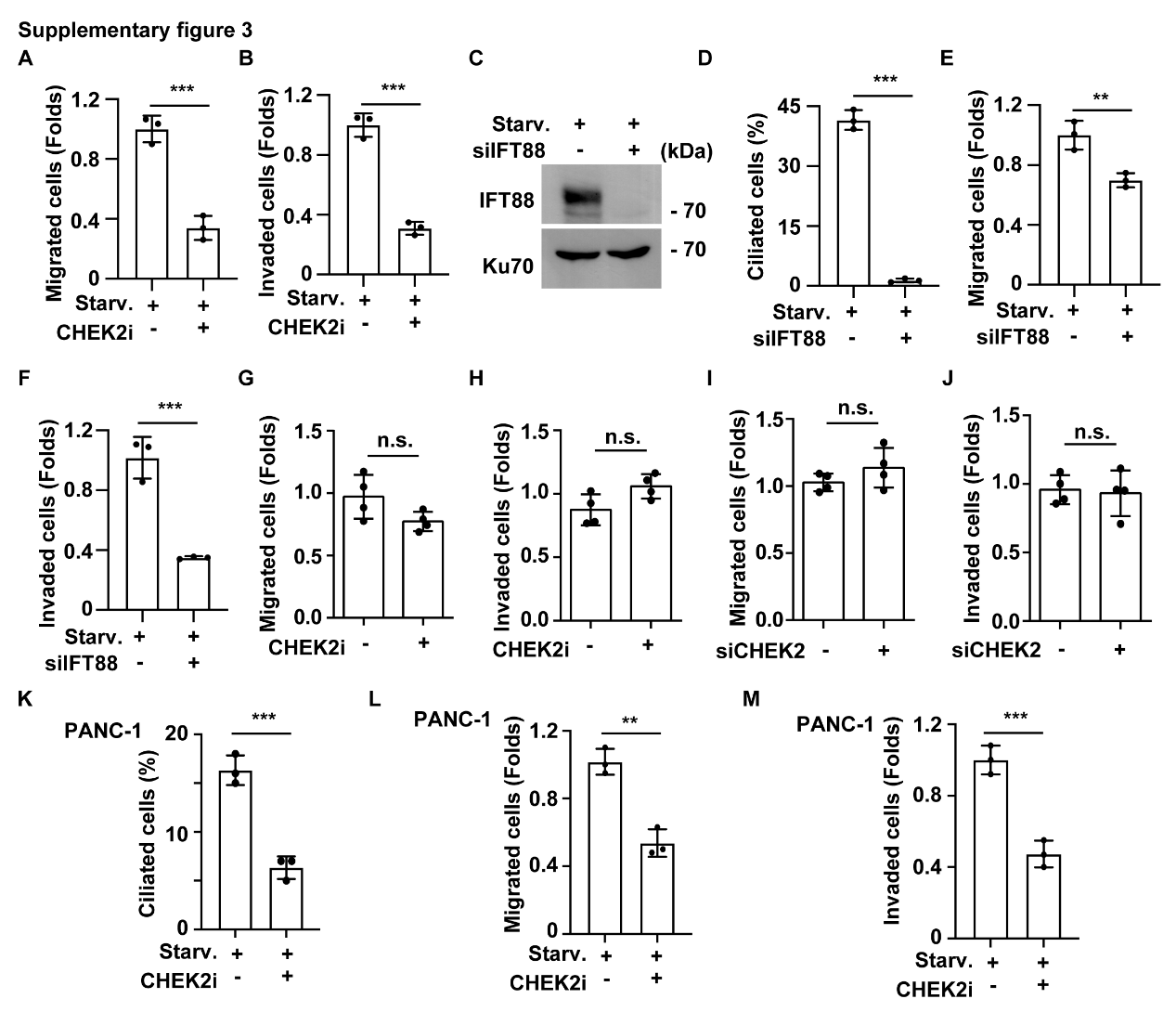


**Supplementary Figure S3. Depletion of IFT88 alleviates cell migration and invasion.**

(**A-B**) Inhibition of CHEK2 reduced trophoblast cell migration and invasion. Quantitative results of relative (A) migrated or (B) invaded cells in CHEK2-inhibited (CHEK2i) HTR-8 cells. (**C-F**) Depletion of IFT88 inhibited primary cilia, cell migration, and invasion. (C) IFT88 was depleted efficiently. Extracts of cells transfected with siRNA against *IFT88* were analyzed by Western blotting with antibodies against IFT88 and Ku70 under serum starvation (Starv.). Reduced numbers of (D) ciliated, (E) migrated, or (F) invaded HTR-8 cells in the presence of *siIFT88* under serum starvation. (**G-J**) Inhibition or depletion of CHEK2 affected trophoblast cell migration or invasion. Quantitative results of (G and I) cell migration or (H and J) invasion in HTR-8 cells treated with CHEK2i (G-H) or *siCHEK2* (I-J). (**K-M**) Inhibition of CHEK2 reduced PANC-1 cell ciliation, migration, and invasion. Quantitative results of (K) ciliated, (L) migrated, and (M) invaded cells. **p<0.01, ***p<0.001. These results are mean ± SD from three independent experiments.

Supplementary Figure S4


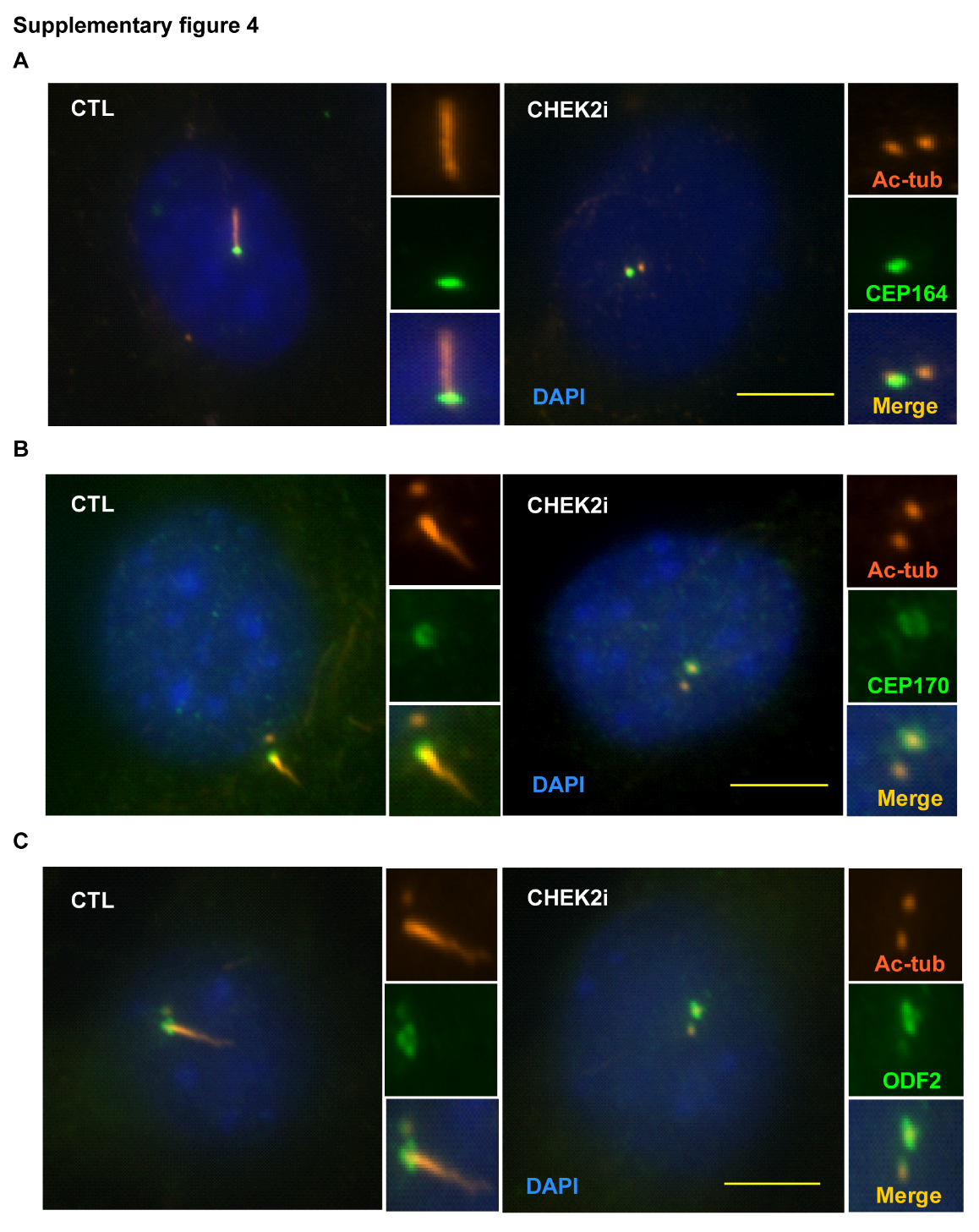


**Supplementary Figure S4. Inhibition of CHEK2 does not affect centriolar appendages.**

(A-C) Centriolar appendages of RPE-1 cells in the absence (CTL) or presence of CHEK2 inhibitor (CHEK2i) were examined by immunofluorescence staining with antibodies against acetylated tubulin (Ac-tub), CEP164, CEP170, and ODF2. Scale bars: 10 μm. DNA were stained with DAPI.

Supplementary Figure S5


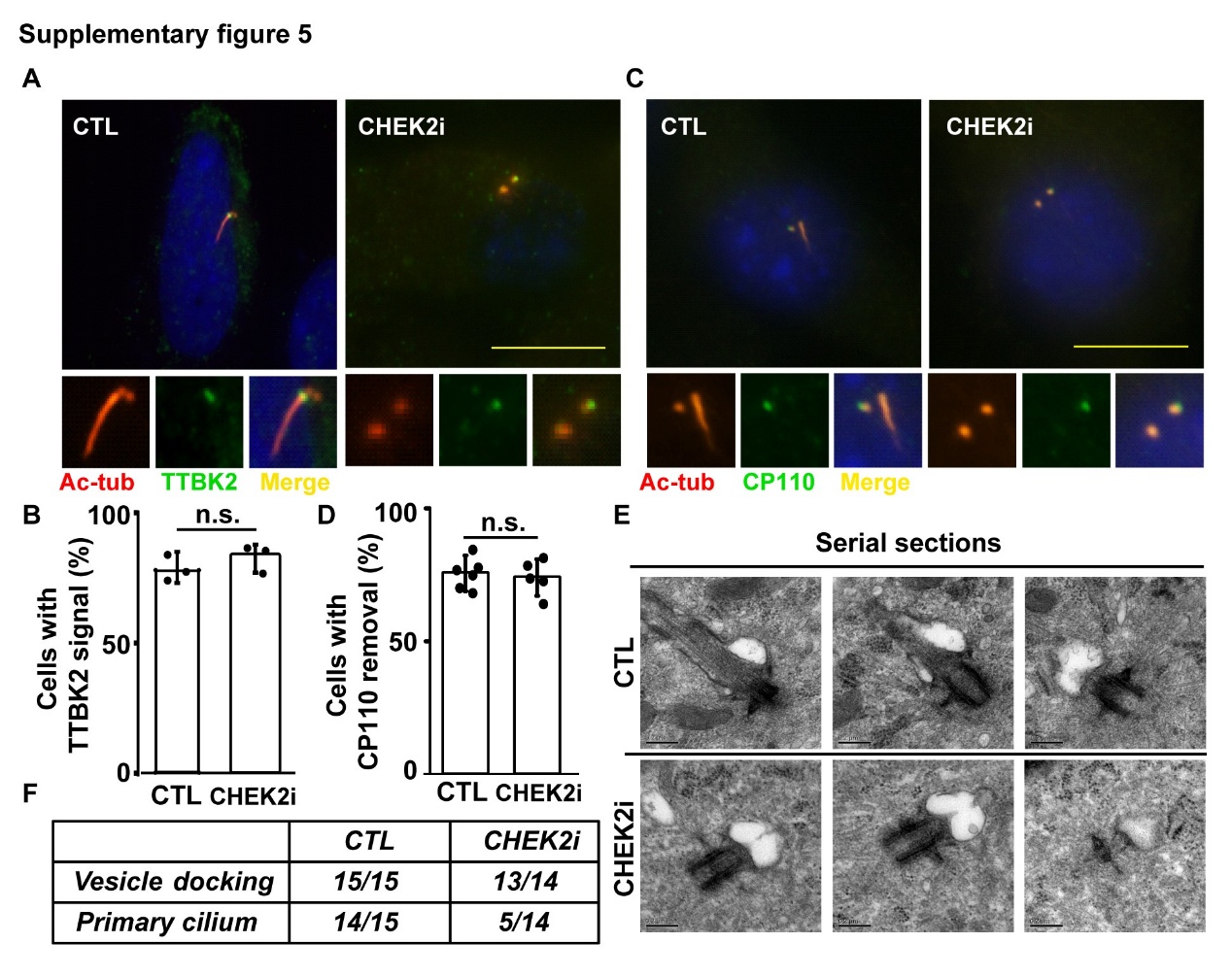


**Supplementary Figure S5. CHEK2 does not affect the initiation of primary ciliation.**

(**A-D**) The inhibition of CHEK2 does not affect TTBK2 recruitment or CP110 removal. (A) Recruitment of TTBK2 to mother centriole in the absence or presence of CHEK2 inhibitor (CHEK2i) was examined by immunofluorescence staining with antibodies against TTBK2 and acetylated tubulin (Ac-tub). Scale bar: 10 μm. DNA were stained with DAPI. (B) Quantitative results of (A). (C) CP110 removal in the absence or presence of CHEK2 inhibitor (CHEK2i) was examined by immunofluorescence staining with antibodies against CP110 and acetylated tubulin (Ac-tub). (D) Quantitative results of (C). (**E-F**) Inhibition of CHEK2 did not affect the docking of ciliary vesicles. (E) Ciliary vesicle docking was examined using transmission electron microscopy with serial sections. CTL: vehicle control. (F) Quantitative results of RPE1 cells with primary cilia and ciliary vesicles in the control (CTL) or CHEK2-inhibited (CHEK2i) cells.

Supplementary Figure S6


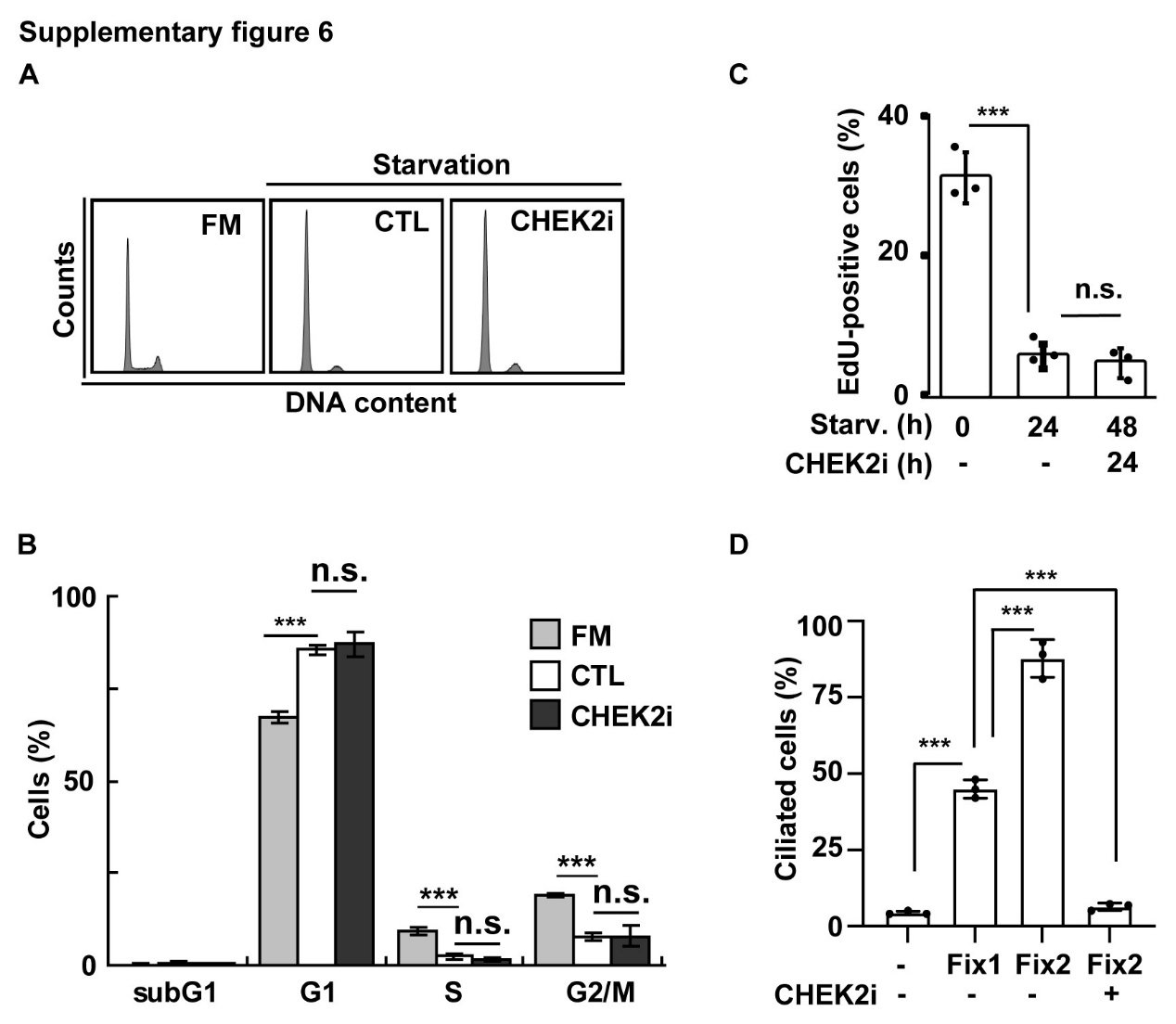


**Supplementary Figure 6. Inhibition of CHEK2 does not disrupt cell cycle arrest caused by serum starvation.**

(**A-B**) Inhibition of CHEK2 did not disrupt serum starvation-induced G1 arrest. (A) Cell cycle profiles of cells cultured in full-medium (FM) or serum starvation in the absence (CTL) or presence of CHEK2i. (B) Quantitative results of (A). (**C**) Inhibition of CHEK2 did not disrupt serum starvation-induced G1 arrest. Quantitative results of EdU-positive cells cultured in serum-starved medium (Starv.) for 24 or 48 h in the absence or presence of CHEK2 inhibitor (CHEK2i). (**D**) CHEK2 maintains primary cilia. Quantitative results of ciliated cells. n.s.: no significance, ***p<0.001. These results are mean ± SD from three independent experiments.

Supplementary Figure S7


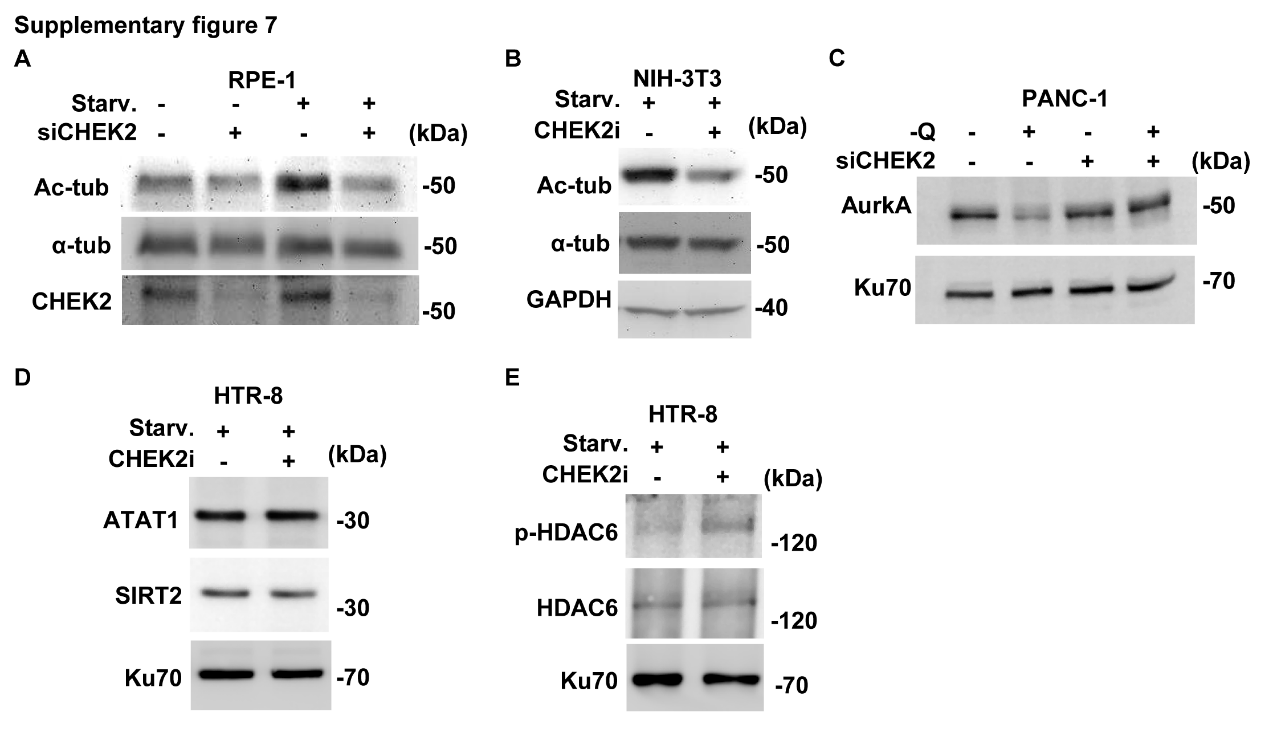


**Supplementary Figure S7. CHEK2 promotes tubulin deacetylation.**

(**A-B**) Depletion or inhibition of CHEK2 reduced tubulin acetylation under serum starvation. Extracts of (A) CHEK2-depleted RPE-1 cells or (B) CHEK2-inhibited NIH-3T3 cells in the absence or presence of serum starvation were analyzed by Western blotting with antibodies against acetylated tubulin (Ac-tub), α-tubulin (α-tub), CHEK2, and Ku70. (**C**) Depletion of CHEK2 restores AurkA under Gln deprivation (-Q). Extracts of PANC-1 cells treated with or without siRNA against *CHEK2* (siCHEK2) were analyzed by Western blotting with antibodies against AurkA, and Ku70. (**D-E**) Inhibition of CHEK2 increased HDAC6 phosphorylation in HTR-8 cells under starvation. (D) Inhibition of CHEK2 did not affect the expressions of ATAT1 and SIRT2. Extracts of serum-deprived HTR-8 cells in the absence or presence of CHEK2i were analyzed by Western blotting with antibodies against ATAT1, SIRT2, and Ku70. (E) Extracts of serum-deprived HTR-8 cells in the absence or presence of CHEK2i were analyzed by Western blotting with antibodies against phosphorylated HDAC6 (p-HDAC6), HDAC6, and Ku70.

Supplementary Figure S8


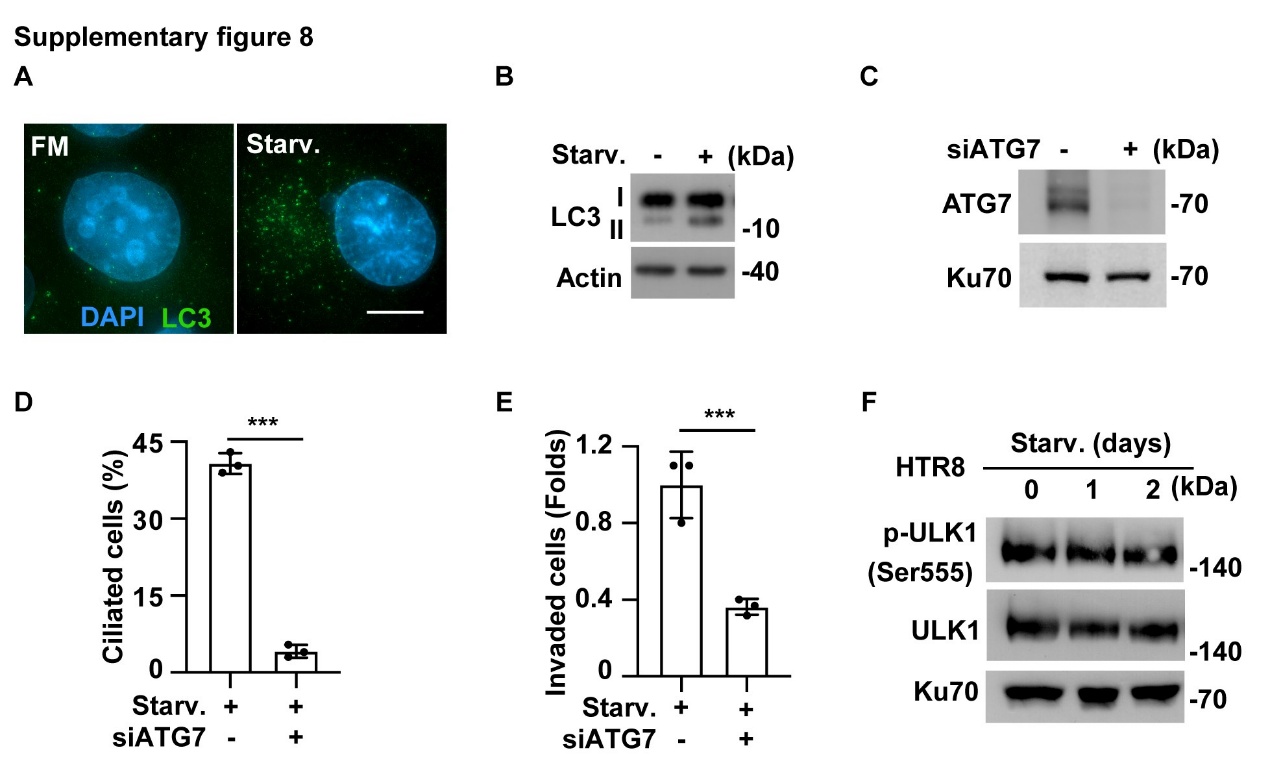


**Supplementary Figure S8. Autophagy contributes to primary cilia-mediated cell invasion.**

(**A-B**) Autophagy was activated in trophoblast HTR-8 cells during serum starvation (Starv.). (A) Increased LC3 immunostaining was detected in cells cultured in full-medium (FM) or serum-starved (Starv.) medium. DNA were stained with DAPI. Scale bar: 10 μm. (B) LC3 II to I ratio increased during serum starvation. Extracts of cells cultured in full-medium or serum-starved medium were analyzed by Western blotting with antibodies against LC3 and actin. (**C-E**) Depletion of ATG7 alleviated primary cilia upon Gln deprivation. (C) ATG7 was depleted efficiently. Extracts of cells transfected with siRNA against *ATG7* (siATG7) were analyzed by Western blotting with antibodies against ATG7 and Ku70. (D-E) Inhibition of AMPK reduced primary cilia and trophoblast cell invasion. (**F**) ULK1 was not activated during serum starvation. Extracts of serum-starved HTR-8 cells were analyzed by Western blotting with antibodies against phosphorylated ULK1 (p-ULK1), ULK1, and Ku70. ***:p<0.001. These results are mean ± SD from three independent experiments.

Supplementary Figure S9


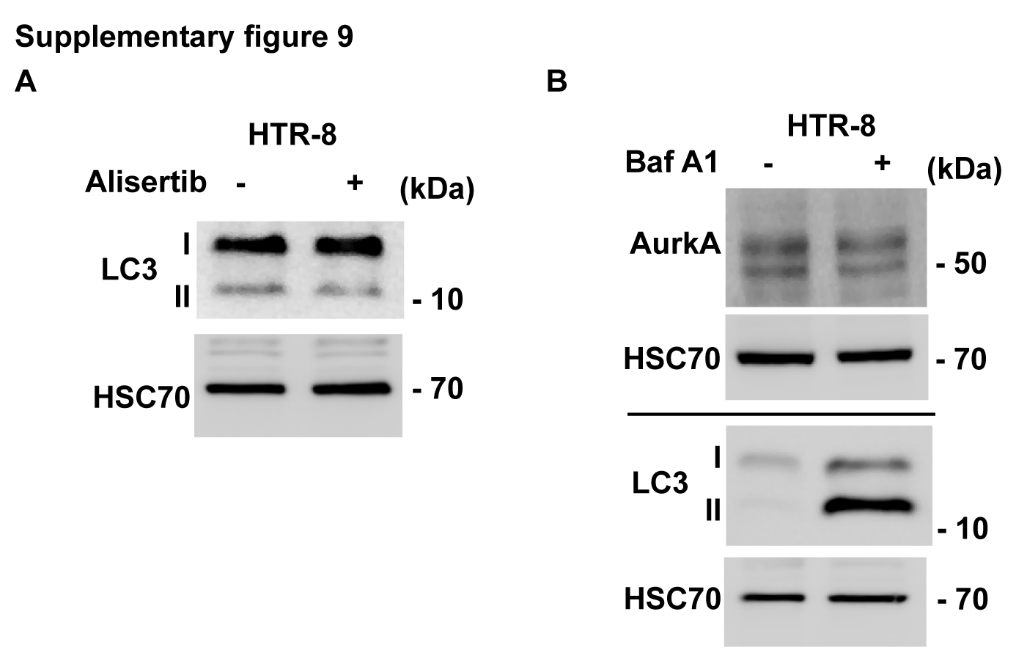


**Supplementary Figure S9. Inhibition of AurkA did not affect autophagy and vice versa.**

(**A**) Inhibition of AurkA did not affect autophagy. Extracts of HTR-8 cells treated with Alisertib were analyzed by Western blotting with antibodies against LC3 and HSC70. (**B**) Inhibition of autophagy did not affect AurkA expression. Extracts of HTR-8 cells treated with Bafilomycin A1 (Baf A1) were analyzed by Western blotting with antibodies against AurkA, LC3, and HSC70.
