# Supplementary figures and images for: Checkpoint kinase 2 coordinates autophagy activation and Aurora kinase A degradation to regulate primary cilia for cell invasion
Source: Cell Commun Signal. 2026 May 22;24:401. doi: 10.1186/s12964-026-02953-6 (PMC13371562; doi:10.1186/s12964-026-02953-6)

Supplementary data

Original blots


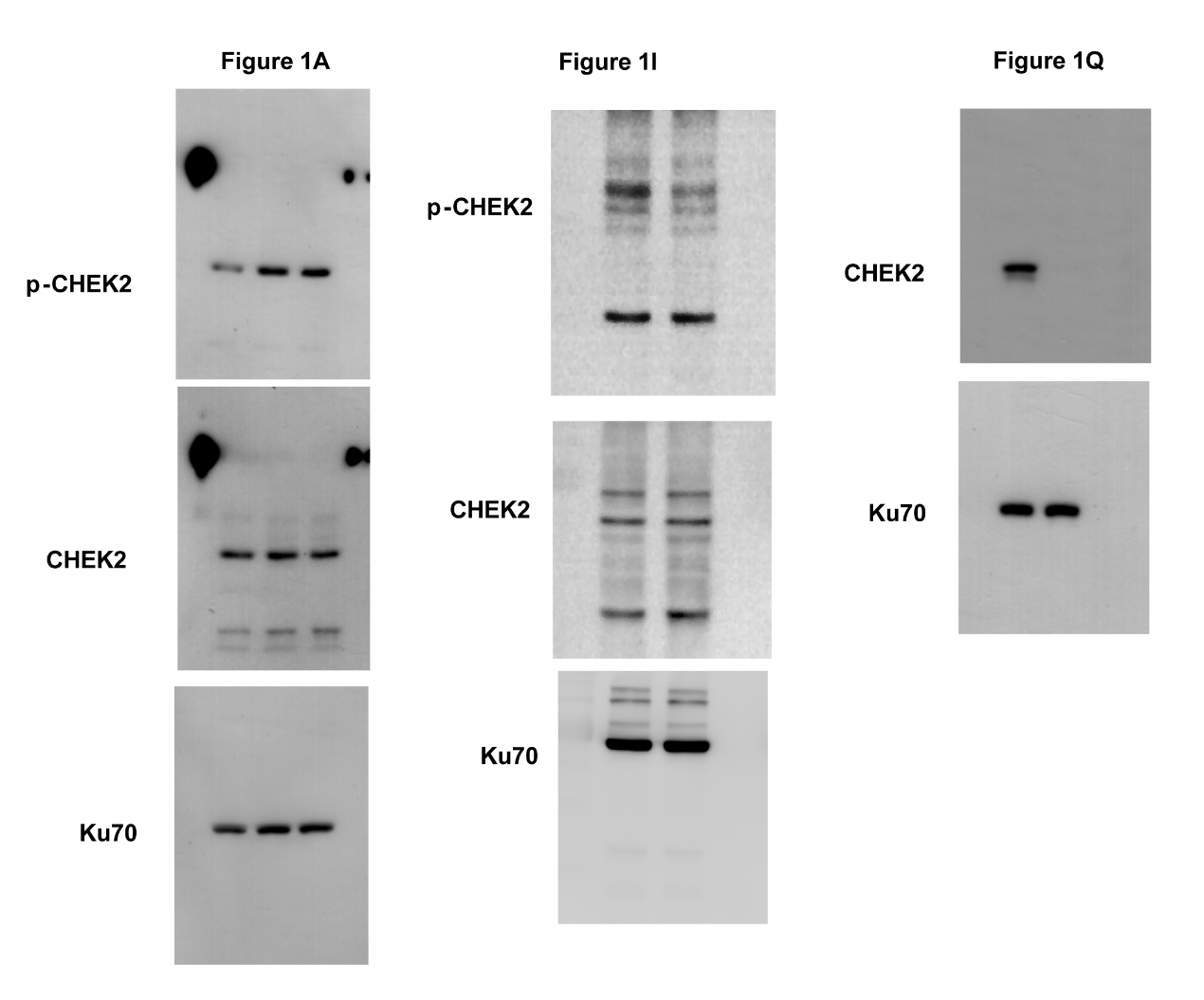


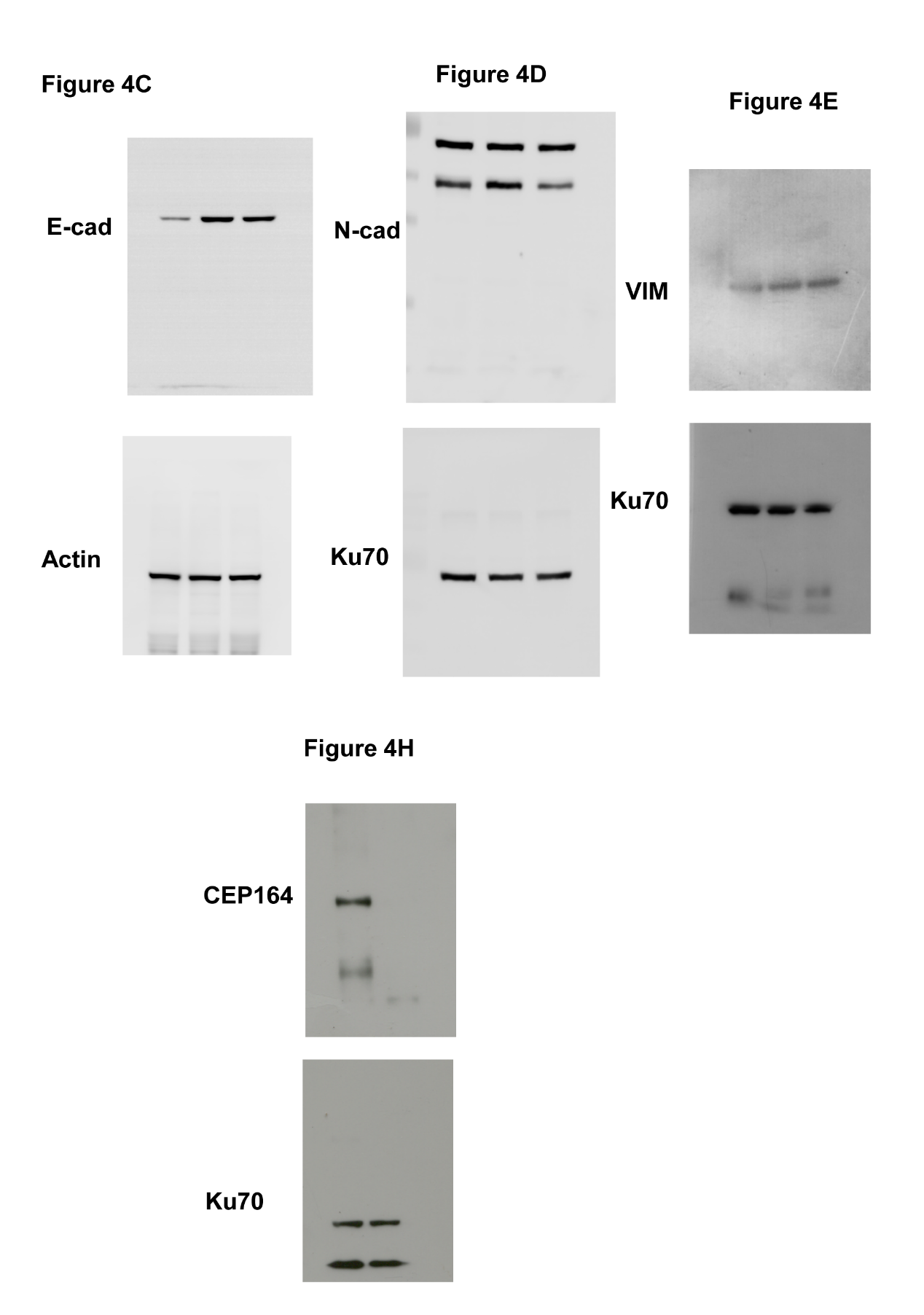


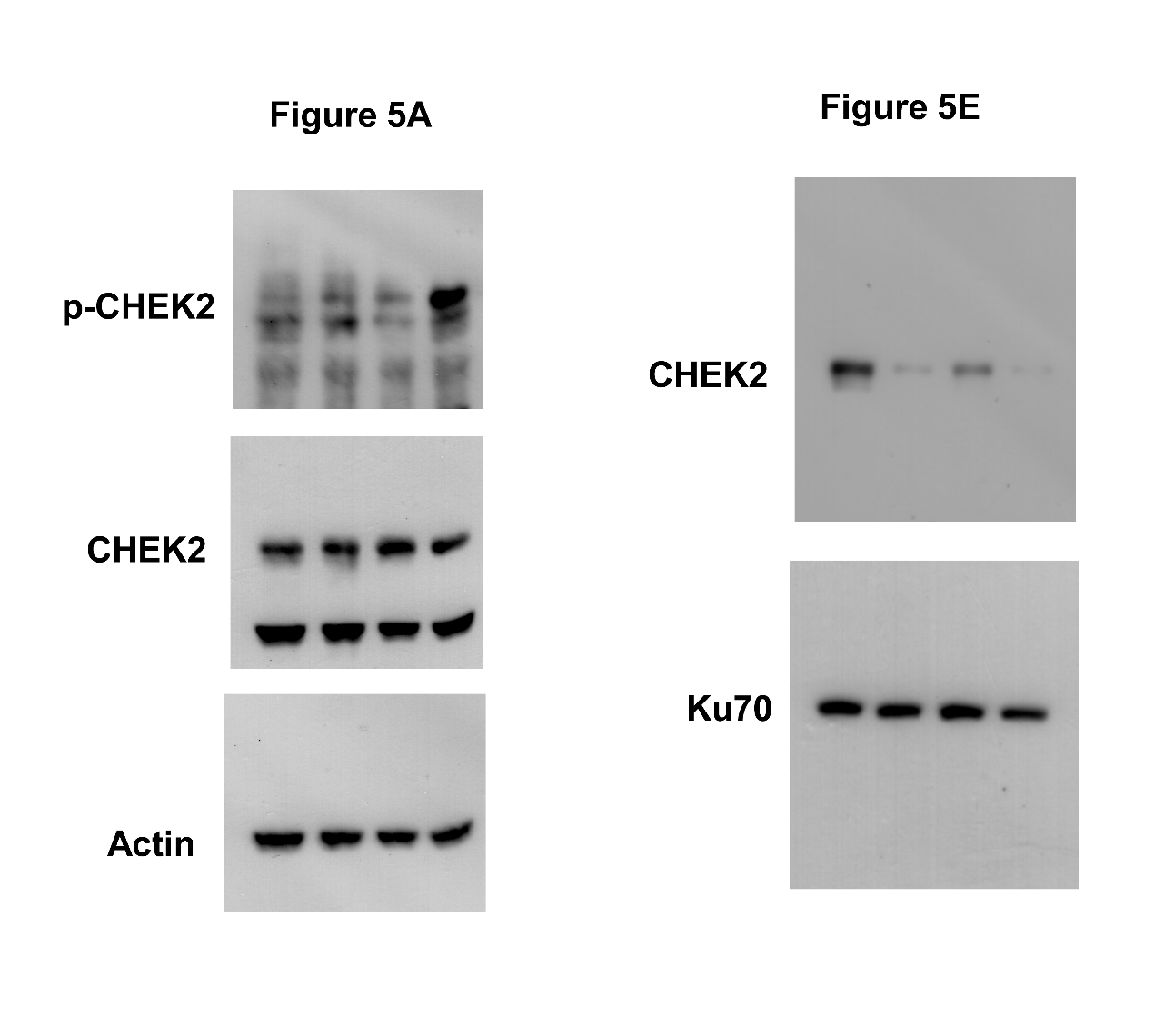


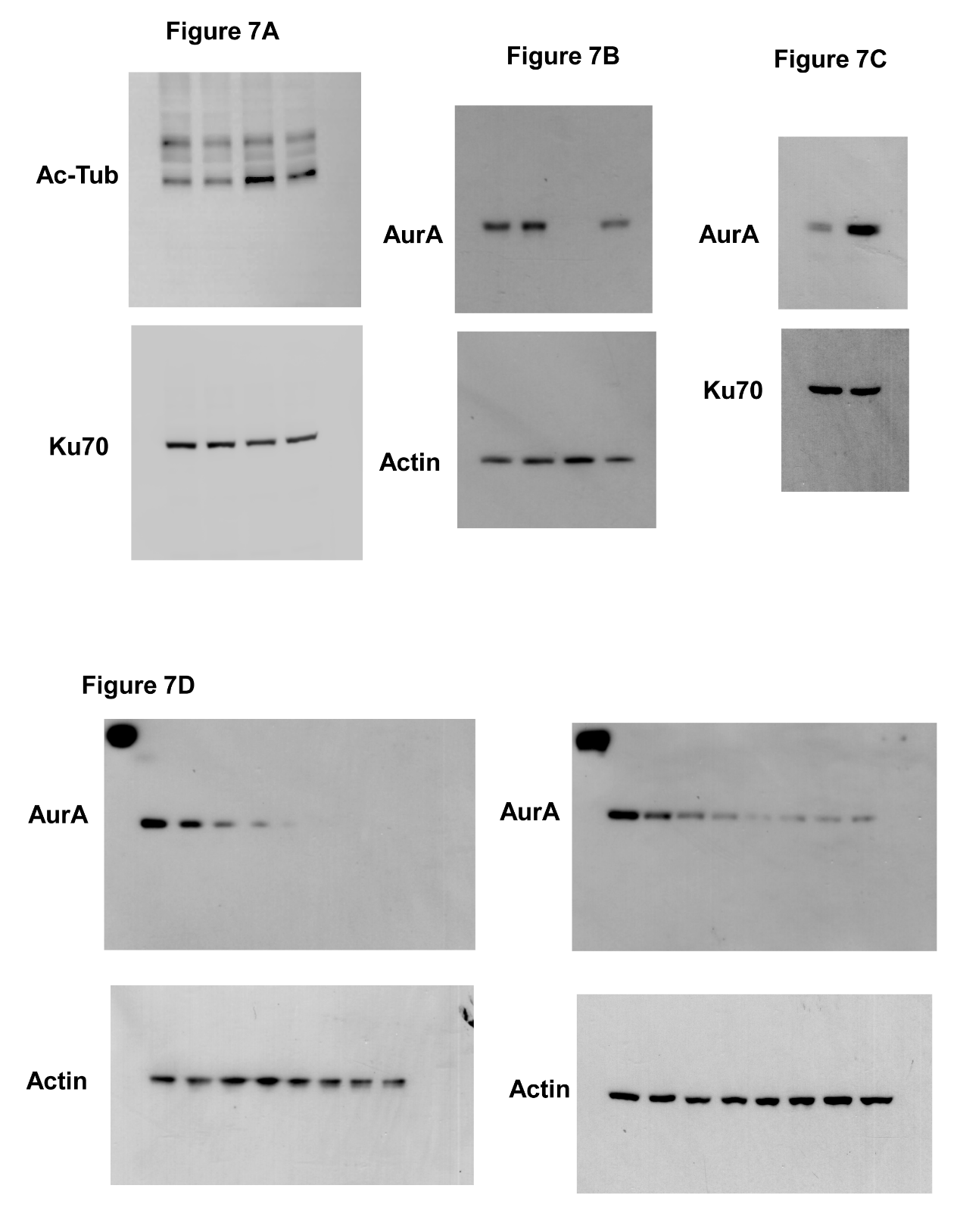


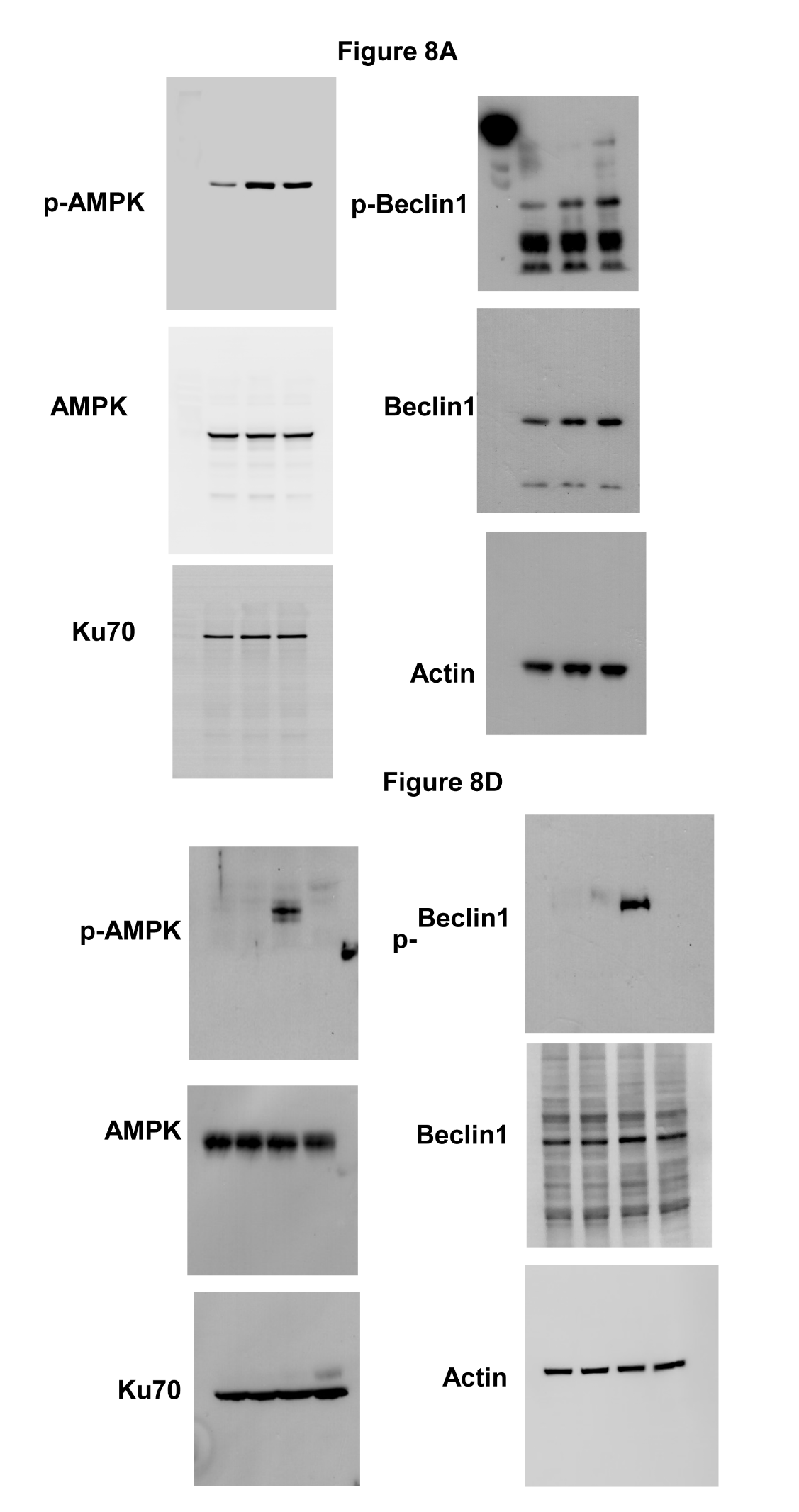

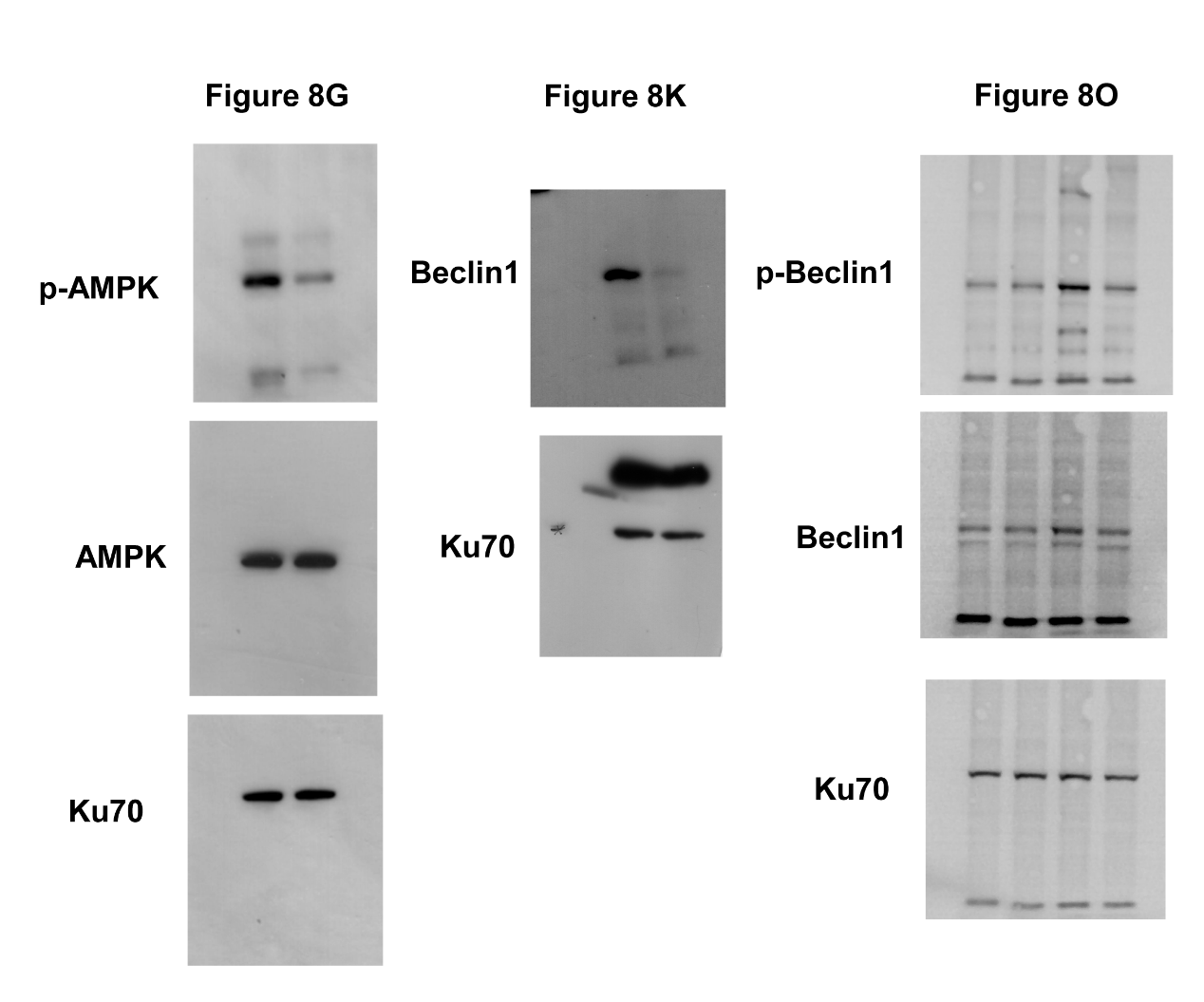

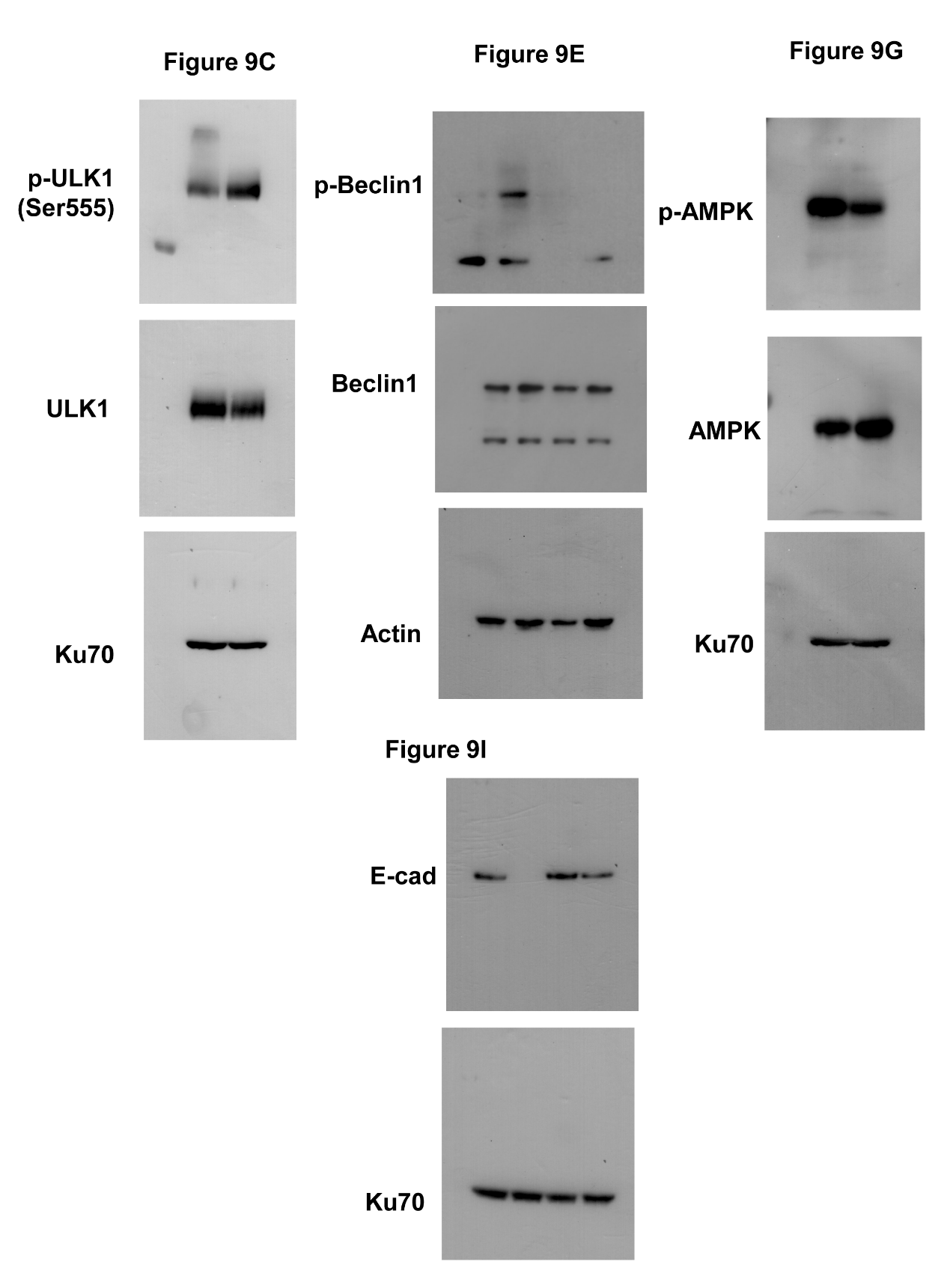


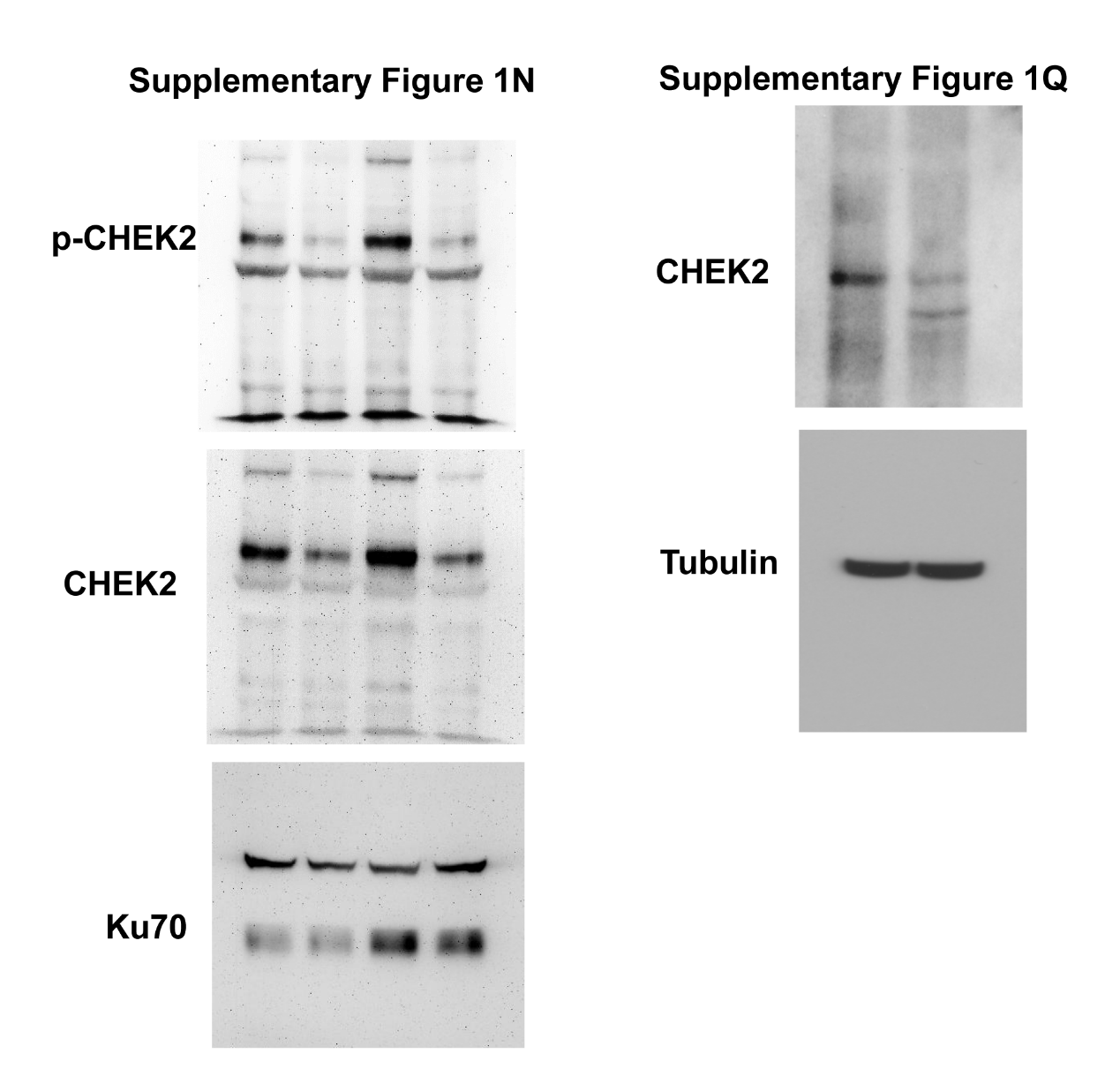


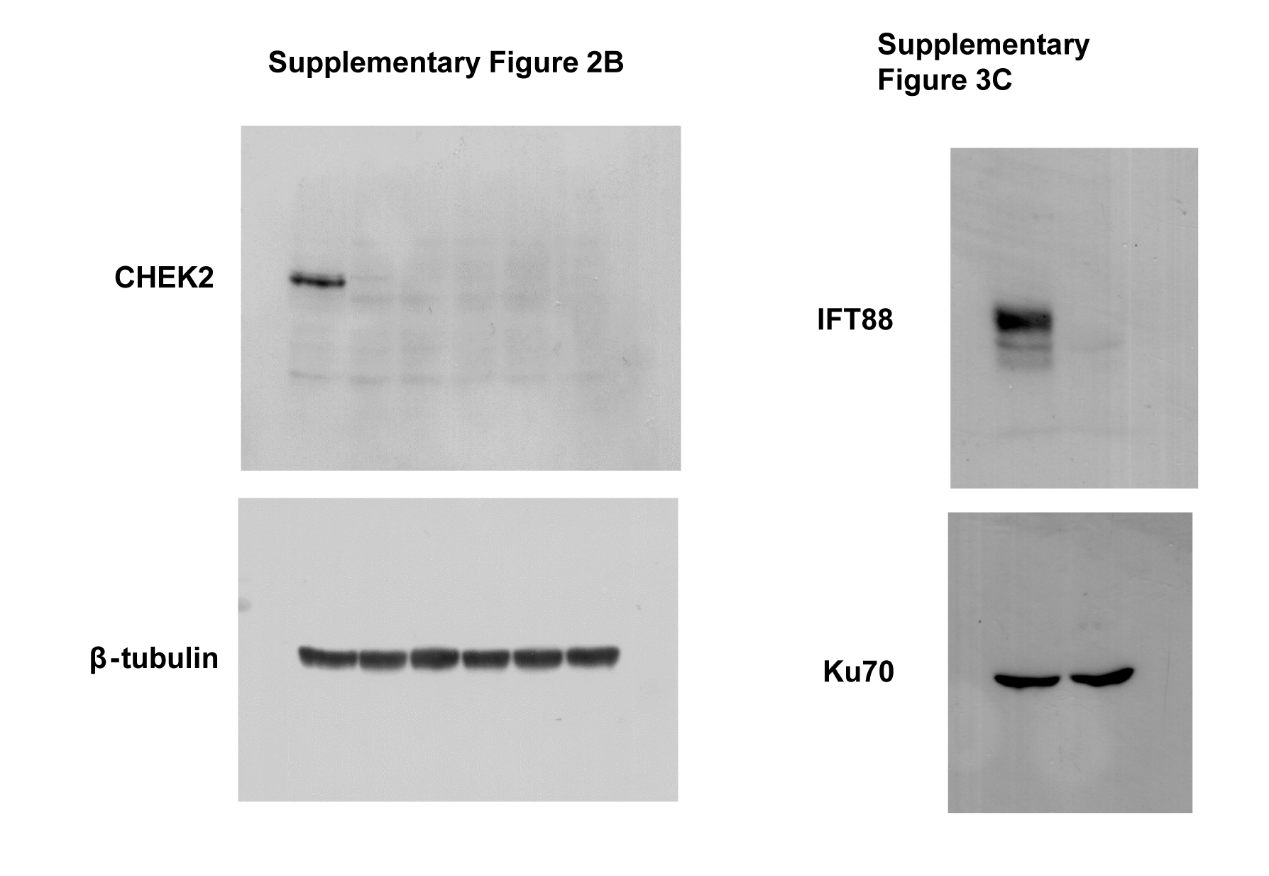


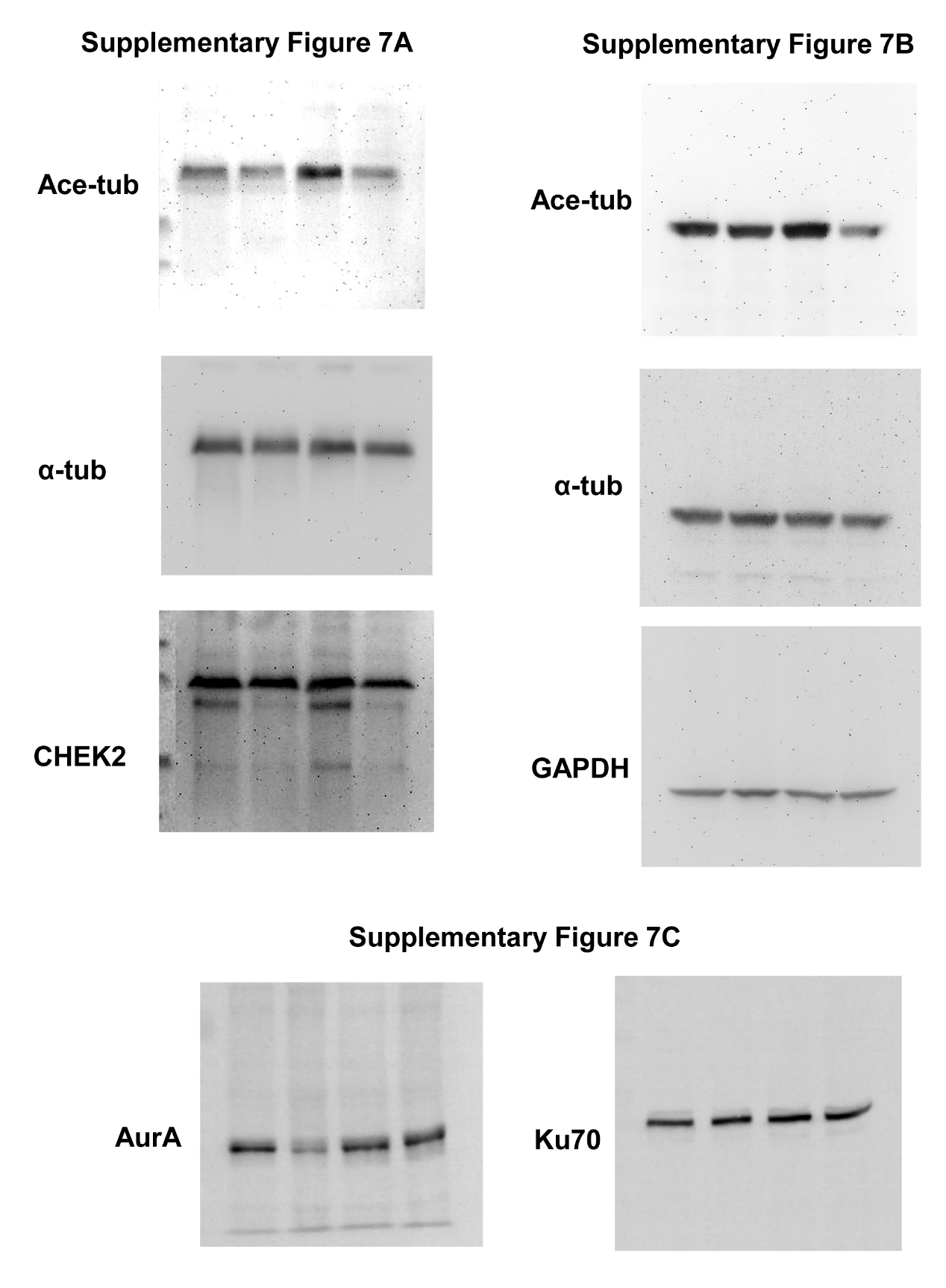


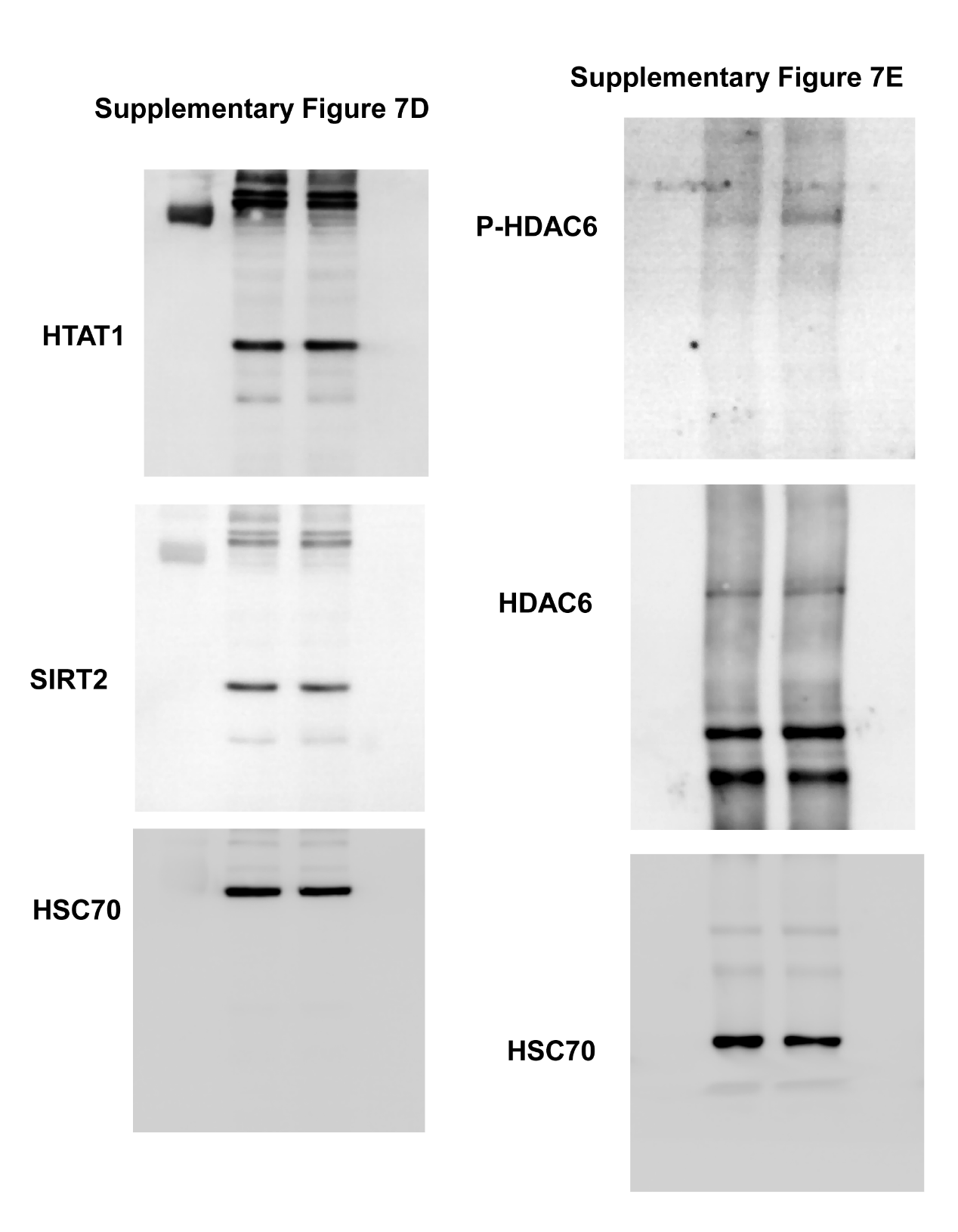


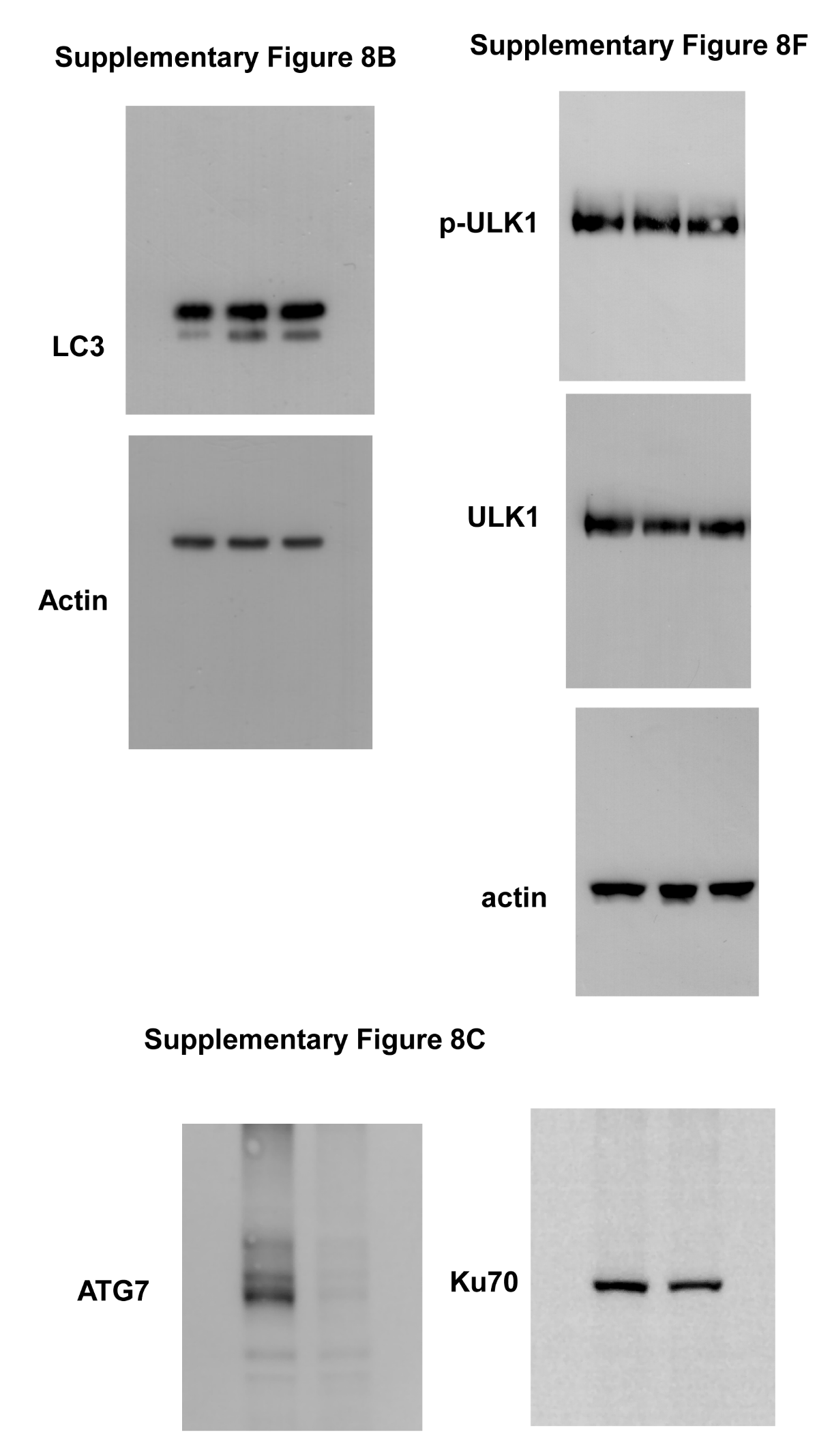


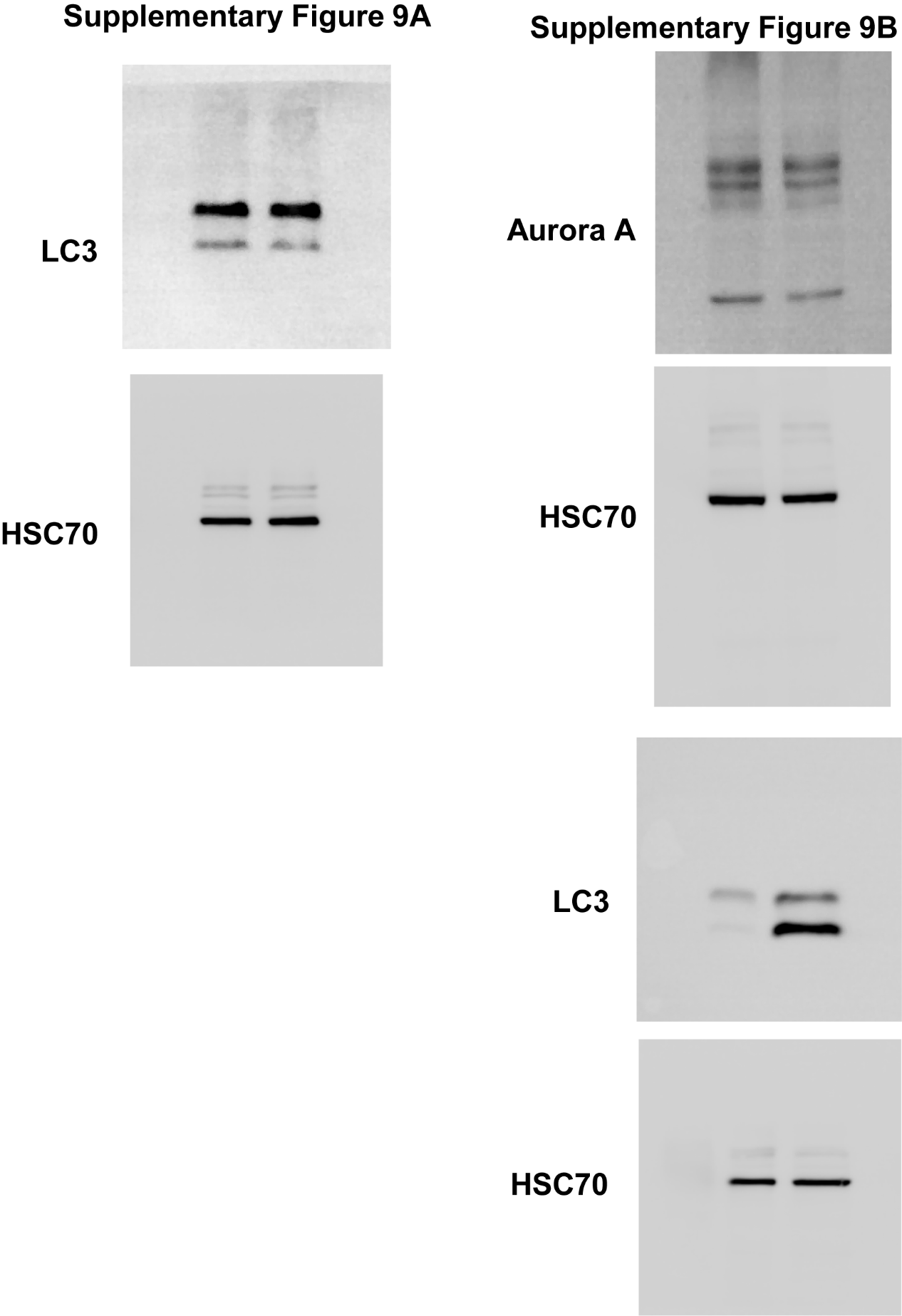

Supplement: Supplementary file 2 — Supplementary Material 2. [file 12964_2026_2953_MOESM2_ESM.docx]
